# Removal of Fat from Surfaces by Lipase-Enhanced Purified Water

**DOI:** 10.1021/acsomega.5c12931

**Published:** 2026-03-16

**Authors:** Andriani Tsompou, Dorina Kokrehel, Kayleigh Davies, Vitaly Kocherbitov

**Affiliations:** 1 Department of Biomedical Science, Malmö University, Malmö SE-20506, Sweden; 2 Biofilms Research Center for Biointerfaces, Malmö University, Malmö SE-20506, Sweden

## Abstract

Growing concern over the environmental impact of conventional detergents has started the search for ecofriendly washing methods, including the use of purified water. Previous studies show that purified water facilitates the removal of olive oil from hydrophilic surfaces via roll-up and bulk mechanisms by promoting electrostatic interactions, while pH-adjusted purified water is effective on hydrophobic surfaces. Here, we investigated lipase-enhanced cleaning with purified and nonpurified water, as well as phosphate buffer solutions, for the removal of olive oil films from both hydrophobic and hydrophilic surfaces, as well as glycerol dioleate films from hydrophilic surfaces. The lipase used was a fungal lipase extracted from *Rhizopus niveus*. Cleaning efficiency was evaluated by gravimetric analysis and quartz crystal microbalance with dissipation (QCM-D). Both methods showed that lipase enhanced cleaning performance in purified water and buffer solution below 40 °C. Even small amounts of lipase in purified water or buffer increased washing efficiency or led to nearly complete oil removal, whereas lipase in tap water showed limited effectiveness. Contact angle and interfacial tension measurements show that the interaction between olive oil and lipase lowers surface tension by hydrolyzing triglycerides into amphiphilic free fatty acids (FFAs). These FFAs act as surfactants at the air/water interface, reducing interfacial tension and promoting oil droplet formation rather than oil spreading, thereby facilitating oil removal. Overall, lipase enables efficient cleaning under mild, surfactant-free conditions, demonstrating strong potential for the development of enzyme-based, environmentally sustainable washing systems based on purified water.

## Graphical abstract

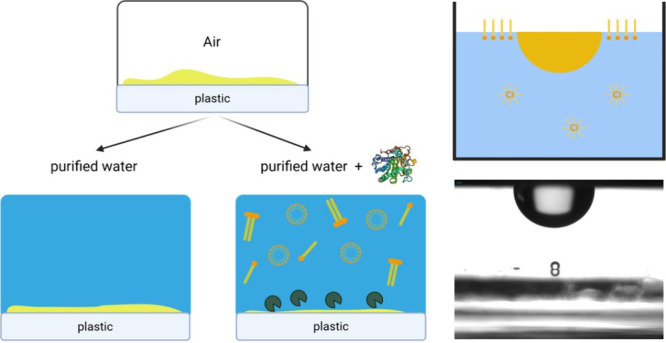

## 1 Introduction

Surfactants are essential in modern detergents, as they lower the surface tension of water and initiate the formation of micelles, allowing hydrophobic soils to be effectively removed during washing.^1^ However, their widespread use presents environmental and health risks. Recent research reveals that surfactants, for example, anionic types like linear alkylbenzenesulfonates (LAS), continue in waste streams and have harmful impacts on aquatic life by disrupting microbial communities.^2–6^ Moreover, surfactants have been found to be linked with skin irritation and respiration in individuals upon prolonged use.^7,8^ As a result, research is turning toward sustainable alternatives.

One proposed solution for a more environmental washing system is the use of ultrapure water, which has shown effectiveness in removing selected soils from hydrophilic surfaces.^9,10^ Alongside this, alternative cleaning methods such as enzymes, natural and degradable, are now researched for their effectiveness in degrading organic residues effectively, in surfactant-free, low-temperature systems.^11–15^ The shift toward lower washing temperatures, driven by environmental care considerations, has made the removal of fatty soils even more difficult by using conventional detergents. Fat-based residues such as butter, cooking oil, and human sebum are particularly persistent on hard surfaces.^16,17^ These challenges have made enzymes increasingly valuable in both laundry and dishwashing applications.^18,19^

Enzymes are widely incorporated into modern detergent formulations because of their ability to degrade soil types. As an example, proteases hydrolyze protein-based stains (e.g., blood, sweat), amylases cleave starches (e.g., sauces, pasta), and lipases break down fats into glycerol and fatty acids.^20,21^ In addition to their efficacy, these enzymes have shown promising synergy with biosurfactants, enabling the development of environmentally friendly detergent systems that reduce or eliminate the need for synthetic surfactants.^11,12^ While enzyme and surfactant combinations are common, the feasibility of enzyme-only systems remains underexplored and could represent an advancement toward fully biodegradable, residue free cleaning technologies. To shift the research toward this, it is important to investigate the performance of enzymes in purified water systems, without the presence of surfactants. For that, the efficiency of washing with purified water grades alone should be first understood.

Systems where purified water grades remove hydrophobic soil from hydrophilic surfaces have been well studied. Studies have shown that purified water can remove hydrophobic soil from hydrophilic surfaces via surface mechanisms such as roll-up followed by bulk processes such as solubilization. Factors such as temperature and washing cycles influence their performance.^9,10^ However, far less is known about how these mechanisms apply to more challenging scenarios, such as removing hydrophobic soils from hydrophobic surfaces.

Hydrophobic surfaces can be challenging in cleaning applications due to their low surface energy and resistance to wetting, which limit the spreading of aqueous cleaning agents. These surfaces promote strong adhesion of soils such as olive oil, through van der Waals interactions, making both physical and chemical removal less effective.^22^ Recent studies have shown that purified water with an increased pH can remove olive oil from a hydrophilic surface with high efficiency. Alkaline pH promotes the deprotonation of fatty acids in olive oil and facilitates oil removal from surfaces through roll up and surface tension mechanisms.^23^

To explore a more sustainable cleaning strategy, it is of interest to examine whether enzymes from natural sources can be used with purified water to remove olive oil or other lipids from hydrophobic and hydrophilic surfaces. For this study, the fungal lipase extracted from *Rhizopus niveus* was used. Lipases can be found everywhere in nature and are produced by a vast number of organisms including animals, plants, and microorganisms. Most lipases function at lipid/water interfaces, where a movable lid domain controls access to the active site. This lid covers the active site in the inactive state and is essential for controlling the enzyme’s catalytic activity. The lid is kept closed in simple aqueous media, whereas it is partially opened when a hydrophobic layer is encountered.^19,24^ The lid thus controls enzyme activity. Once the active site is open, lipases start to break down the ester bonds of triglycerides and hydrolyze them to mono- and diglycerides, free fatty acids, and glycerol.^18,19,24,25^ The hydrolyzed products are more water-soluble in comparison to intact triglycerides and hence they can more easily be dispersed in aqueous systems.^24^

In this study, we focus on using lipase with different types of solutions (phosphate buffer) and water grades, such as purified (MQ, DIRO) and nonpurified water grades (TAP), in order to remove thick films of olive oil from a plastic hydrophobic surface and thin films of lipids from hydrophilic surfaces using gravimetric analysis and quartz crystal microbalance and dissipation, respectively. Surface and interfacial tension measurements were done to understand how the lipase can promote oil removal. Based on the results and previous research on the topic, we investigated the most efficient and sustainable way of removing hydrophobic soils without the use of detergents.

## 2 Materials and Methods

### 2.1 Materials

Fungal lipase extracted from *Rhizopus niveus* (RNL, Sigma-Aldrich, Germany) was analyzed and evaluated in these experiments. Sodium phosphate (SP) buffers were prepared using sodium phosphate monobasic monohydrate (NaH_2_PO_4_·H_2_O) and sodium phosphate dibasic dihydrate (Na_2_HPO_4_·2H_2_O). pH was adjusted with sodium hydroxide (NaOH) and hydrochloric acid (HCl) (chemicals obtained from Sigma-Aldrich). If not noted otherwise, the SP buffer solutions were prepared at pH 7.7.^26^ MQ water was produced at the laboratory of Malmö University, Sweden, using a PURELAB flex (ELGA, UK). DIRO water was provided by SWATAB, Malmö, Sweden. TAP water was collected in the university building. Extra virgin olive oil (FONTANA est. 1978, classic, Spain) and glycerol dioleate (GDO, from Croda Staffordshire, United Kingdom) were used as model fats. 15 mL polypropylene (PP) plastic tubes (SARSTEDT, Germany) were used as a hydrophobic substrate.

### 2.2 Conductivity and pH Measurements

The conductivity of SP buffers and different water grades, both with and without RNL (0.001–0.1 wt %), was measured using a Bench conductivity/TDS Meter CON 510 (Eutectic instruments, Singapore). Each solution was measured individually for a duration of 5 min, with conductivity values recorded at 1 min intervals, resulting in five data points per solution. The conductivity electrode was rinsed with MQ water between measurements to prevent cross-contamination.

The pH of the same SP buffer solutions and water grades (with and without 0.001–0.1 wt % RNL) was determined using a 744 pH Meter (Metrohm, Denmark). The pH electrode was rinsed with MQ water after each measurement. Single-point measurements were taken for each solution.

### 2.3 Zeta Potential

The isoelectric point (pI) of the RNL was determined via zeta potential measurements. Two sets of 0.1 M SP buffer solutions were prepared across a pH range of 2 to 8. RNL was added to achieve a final concentration of 0.01 and 0.1 wt % in each set. Zeta potential was measured by using a Zetasizer Ultra instrument (Malvern Panalytical, UK) with folded capillary zeta cells (DTS0012). Prior to measurements, the cells were rinsed with MQ water, ethanol, and MQ again to ensure the cleanliness. Data acquisition and analysis were carried out using the ZS XPLORER software (Malvern Panalytical, UK).

### 2.4 Gravimetric Analysis: Measurement of Oil Film Mass before and after Water Contact

Gravimetric analysis was conducted to evaluate the effectiveness of various water grades and sodium phosphate buffer solutions, with and without the addition of lipase (RNL), in cleaning hydrophobic surfaces. Three RNL concentrations were tested, ranging from 0.0001 to 0.01 wt %. Empty plastic tubes (15 mL, 120 × 17 mm; transparent polypropylene, Sarstedt, Nümbrecht, Germany) were weighed using an AT261 Delta Range analytical balance (Mettler-Toledo, Columbus, x C, USA). Approximately 14–15 mg of olive oil was applied to each tube and evenly spread using a cotton swab. 5 mL of washing solution was added to each tube. Tubes were rotated at 26 rpm by using a tube revolver rotator (Thermo Fisher Scientific, Massachusetts, USA) to perform the washing process. After washing, excess water was discarded. The tubes were freeze-dried overnight in a benchtop Alpha 1–4 LSC system (VWR International, Radnor, USA) at −60 °C and 0.080 mbar. The following day, the mass of the tubes was measured.

Following the procedure described above, the tubes were washed using various water grades and SP buffer solutions (0.025–0.1 M) at 25 °C for 1, 5, 30, and 60 min to determine the optimal washing time of each cycle. To determine how different temperatures and different washing cycles affect the washing efficiency, the tubes were washed at 25 and 40 °C for 4 washing cycles.

The washing efficiency was determined based on the mass difference between the unwashed and washed samples. First, the initial mass of the surface was recorded prior to washing. After the washing procedure and drying of the sample were performed, the final mass was measured. The mass of the soil removed was calculated as the difference between the initial and final masses. Washing efficiency (%) was then expressed as the ratio of the removed mass to the initial mass multiplied by 100.

### 2.5 Quartz Crystal Microbalance and Dissipation (QCMD)

All experiments were performed using a Q-Sense QCM-D E4 unit equipped with a standard flow module (Biolin Scientific AB, Sweden). The sensors used for the experiments are the QSX 303 SiO_2_ QCM-D (5 MHz) sensors from Biolin Scientific AB (Sweden). All sensors were cleaned in accordance with the manufacturer’s recommendations. For the film, glycerol dioleate (GDO) from Croda Staffordshire, United Kingdom, was used. All measurements were performed and were measured at 25 °C.

The films were deposited on the sensor by spin coating. To coat the sensors, the following procedure was used: GDO was dissolved in ethanol (5 mg/mL). 20 μL of the sample was deposited on the sensor surface while the sensor was rotated in 1200 rpm (the spin-coater was developed in house). The sensor was left to dry in the fume hood for 15 min and overnight in a desiccator.

To calculate the washing efficiency, each sample was examined in air before and after contact with liquid. The main source of errors for measurements of thin films was mounting and remounting of the sensor in the QCM-D module, which results in a shift of the resonance frequency and introduces an error of ± 20 Hz.^9^ To eliminate this inaccuracy, all measurements in air were performed five times.

For the complete QCM-D protocol, the bare sensor was initially measured in air five times, with each measurement lasting 5 min. The same sensor was then spin-coated and subsequently measured five additional times in air. In the next phase, the sensor underwent a washing procedure using all relevant solutions. These solutions were introduced at a flow rate of 250 μL/min; upon reaching the QCM-D module, the flow rate was reduced to 80 μL/min for 35 min followed by 60 μL/min for 25 min. After the washing process, the sensor was removed from the module and dried with nitrogen gas. The dried and washed sensor was then reinserted into the module and measured in air five times.

A positive control was included in the experiment: this control sensor was washed with 2% SDS in MQ water for 15 min at a flow rate of 250 μL/min followed by a 10 min wash with MQ water at the same flow rate. The thickness of all films was calculated via the Voigt model in QTools. Washing efficiency was calculated following the same approach used in gravimetric analysis, only this time, the washing is expressed as thickness via the Voigt model in QTools.

At each stage, the sensors were examined by using a Nikon Optiphot microscope (Nikon, Melville, USA). Images were captured with the cellSens Entry software (OLYMPUS, Tokyo, Japan).

### 2.6 Contact Angle, Surface, and Interfacial Tension Measurements

Contact angle measurements were performed on both glass slides (VWR Microscope Slides with cut edges, VWR International) and polypropylene slides (RS Group, London, UK). All surfaces were cleaned by rinsing with MQ water, ethanol, and MQ again followed by drying with nitrogen gas. Additionally, glass slides were plasma-cleaned for 15 min using a Plasma Cleaner/Sterilizer PDC-32 G (Harrick, USA) to ensure surface uniformity.

Measurements were carried out using a Drop Shape Analyzer DSA100 equipped with ADVANCE software (KRÜSS, Germany). Contact angles were analyzed using the ADVANCE and ImageJ software.

For the static measurements, each solution (5 μL) was deposited onto the test surfaces and analyzed under ambient conditions. For the measurements in liquids, olive oil droplets (5 μL) were deposited onto the surfaces followed by the addition of 3 mL of each aqueous solution. For time-resolved measurements, 5 μL olive oil droplets were deposited on the surface. 3 mL of the respective aqueous solution was added, and then the contact angle evolution was monitored for 10 min. Images were captured every 30 s. These measurements were conducted on glass slides. The data was collected in triplicate.

Surface and interfacial tension experiments were performed and analyzed with the same equipment.

## 3 Results and Discussion

In this study, gravimetric analysis was used to investigate how RNL can remove olive oil films from hydrophobic surfaces. The effect of the number of washing cycles and their duration was studied. QCM-D was used to understand how RNL lipase can remove thin lipid films from a hydrophilic silica surface, while surface and interfacial tension were used to understand the mechanisms of the removal of hydrophobic soils from hydrophobic surfaces.

### 3.1 Properties of Lipase in Different Solutions

To see how the charge of lipase (Figure 1) changes at different pH levels, the isoelectric point (pI) was determined by zeta potential measurements. As shown in Figure 2a, the net charge switches from positive to negative between pH 2 and 3. From these measurements, the isoelectric point (pI) was calculated as 2.7 on average, based on the results of two independent experiments performed at different lipase concentrations (0.01 and 0.1 wt %). The influence of lipase on the pH was also studied. As summarized in Table S1, the pH of every buffer and water grade remains essentially unchanged after the enzyme is added.

**Figure 1:**
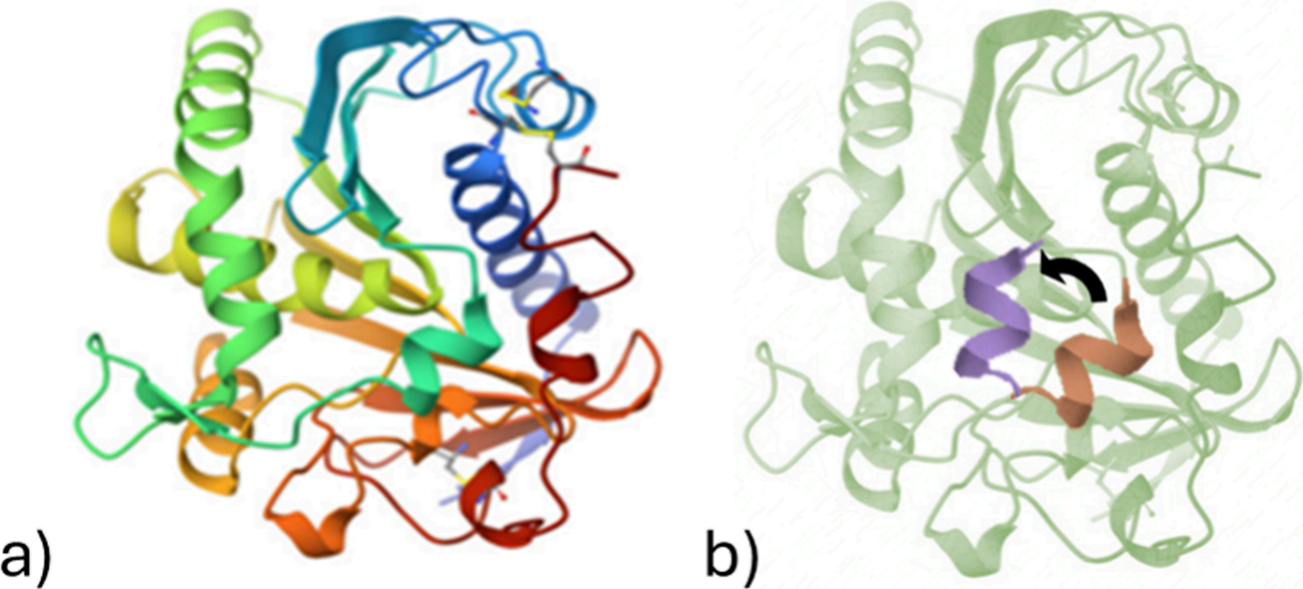
(a) Crystal structure of RNL obtained from the RCSB Protein Data Bank.^27^ (b) RNL highlighting its amphiphilic lid structure. The lid adopts two conformations: orange indicates the closed (inactive) state, and purple represents the open (active) state.

**Figure 2:**
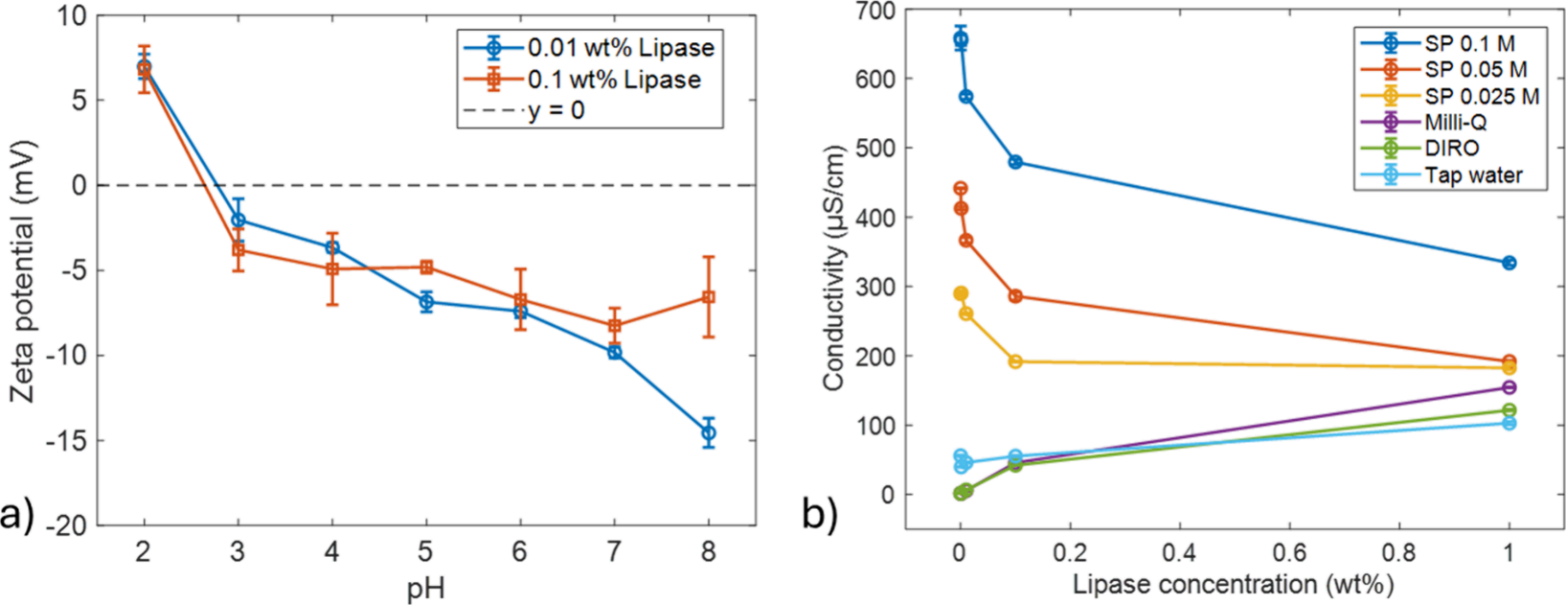
(a) Zeta potential measurements based on 0.01 and 0.1 wt % RNL solutions at different pH values. (b) Conductivity measurements based on RNL concentrations from 0 to 0.1 wt %. Data is plotted as mean values with standard errors. SP buffers and water grades were analyzed.

Since this study involves various water grades and buffer concentrations, it is important to first characterize their ionic properties when lipase is not present. We refer to MQ and DIRO as purified water grades, while TAP is a nonpurified water grade. One difference between purified and nonpurified water is their ionic content and the presence of impurities. These differences are captured by conductivity measurements. As shown in Figure 2b, in the absence of lipase, the conductivity of purified water (MQ and DIRO) is the lowest followed by TAP water, while the sodium phosphate (SP) buffer solutions display progressively higher conductivity as the buffer concentration increases.

Upon addition of RNL, the solution conductivity varies strongly with the ionic environment. In pure water, where the initial concentration of ions is very low, the addition of charged lipase molecules increases the overall number of charges, leading to a slight increase in the conductivity. In the case of buffer solutions that already contain high concentrations of small, highly mobile ions, the conductivity decreases. The negative charge of the lipase (pI is 2.7) at pH 7.7 introduces electrostatic attraction between the negatively charged lipase and the positively charged sodium ions, which can lead to the decrease of the free ions in the bulk. This will be translated into a decrease in the conductivity. Although lipase introduces additional charges into the system, its large size and very low mobility imply that it contributes little to charge transport compared with small ions.^28,29^ More generally, this reflects the principle that while macromolecules can affect ion availability through binding, their direct contribution to conductivity is negligible compared to that of small fast-moving ions.^30^

### 3.2 Effect of Washing Treatment Time on Plastic Tube Cleaning Efficiency

Gravimetric analyses were conducted to determine whether the addition of *Rhizopus niveus* lipase to various water grades enhances their ability to remove olive oil from hydrophobic surfaces. Sodium phosphate buffers were used as controls to evaluate the cleaning efficiency of RNL under conditions reported to be optimal for its activity (pH, ionic strength).^31^

Tubes were washed with different water grades and SP buffer solutions at 25 °C at 4 independent washing times (1, 5, 30, and 60 min) for 1 washing cycle. Figure 3 shows the corresponding results in the absence of lipase. Washing with SP buffers at 0.05 and 0.025 M consistently decreased the remaining mass with washing time. Independent of the washing protocol used, SP in these concentrations achieved the highest cleaning efficiency. Purified water grades showed a small variation of mass with washing time. A different trend was observed for TAP water and the SP buffer at 0.1 M. While both showed an initial oil removal, prolonged washing (60 min) resulted in an increase of the mass, resulting in a reduced efficiency compared to shorter washing cycles.

**Figure 3:**
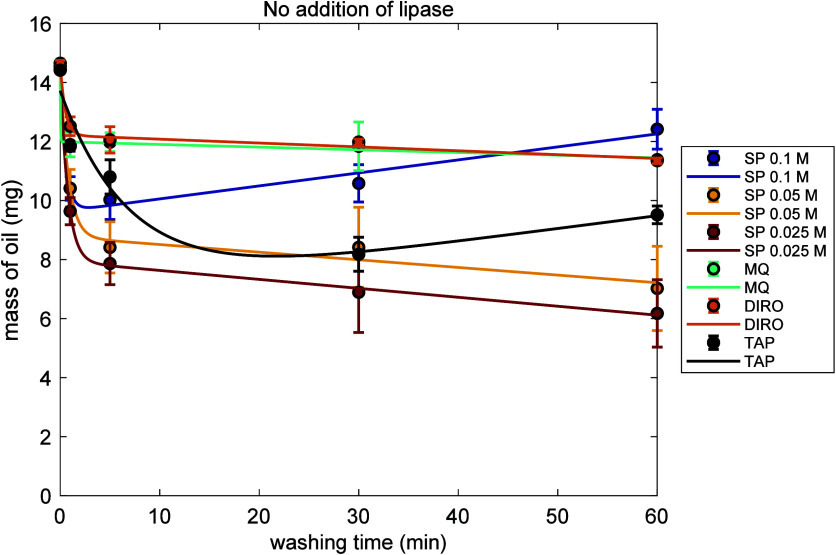
Single cycle washing experiments without lipase. Average amount of olive oil remaining on the surface of plastic tubes (mg) after washing at 25 °C for 1, 5, 30, and 60 min using sodium phosphate buffer at three concentrations: (0.1, 0.05, and 0.025 M) and three different water grades (MQ, DIRO, and TAP). Each condition was tested in triplicate. Data is fitted with eq S1.

Figure 4 shows the washing efficiency of 0.1 and 0.025 M SP buffers with the addition of different RNL concentrations at 25 °C. In both cases, the absence of lipase resulted in the lowest cleaning efficiency, as indicated by the higher remaining residual mass on the surface regardless of washing duration (Figure 4). Washing efficiency improved when the lipase concentration was increased from 0.0001 to 0.001 wt %, while further increasing it to 0.01 wt % produced a similar cleaning effect.

**Figure 4:**
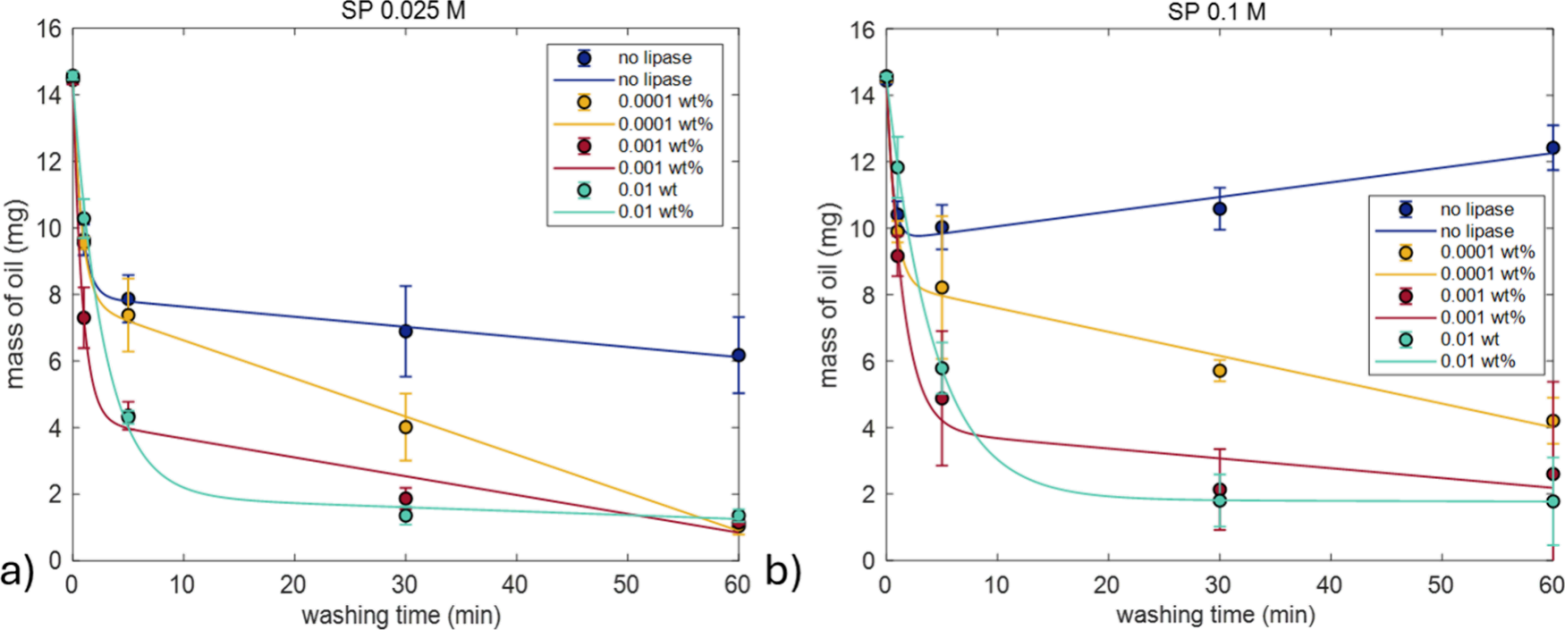
Single-cycle washing experiments in phosphate buffers. Average residual mass remaining on the surface of plastic tubes (mg) after washing at 25 °C for 1, 5, 30, and 60 min using sodium phosphate buffer at two concentrations: (a) 0.025 M and (b) 0.1 M. Results are shown for conditions without lipase (blue) and with lipase (yellow, red, green) dissolved in the respective buffers. Each condition was tested in triplicate. Fitting parameters can be found in Table S3 and plots for SP 0.05 M in Figure S1. Data is fitted with eq S1.

Regarding washing time, short durations of 1 and 5 min reduce the amount of oil in surface, but extending the wash to 30 min enhances cleaning, independent of SP or RNL concentration. For example, a 5 min wash with 0.025 M SP and 0.0001 wt % RNL removed 70.4% of the initial oil, whereas a 30 min wash under the same conditions achieved 90.6% removal, representing a 29% relative increase in efficiency for only a trace amount of enzyme. Figure S1b shows that washing with an SP buffer of 0.05 M in the same concentrations of lipase results in the same washing trend as Figure 4.

When different water grades were used for washing of the plastic surface, the washing efficiency varied (Figure 5). In the absence of lipase, TAP water was able to remove more oil than the purified grades irrespective of the washing time, although the difference is clearer after a 30 and 60 min wash (Figure 3, Figure S2). After a 60 min wash, MQ and DIRO had an efficiency of 21.06 and 22.59%, respectively, while TAP had 33.97%. TAP water’s higher efficiency without addition of lipase is explained by its higher pH. At higher pH, more hydroxide ions are present, which facilitate the saponification and deprotonation of fatty acids into ionized fatty acids that can easily be removed from the surface.^23^

**Figure 5:**
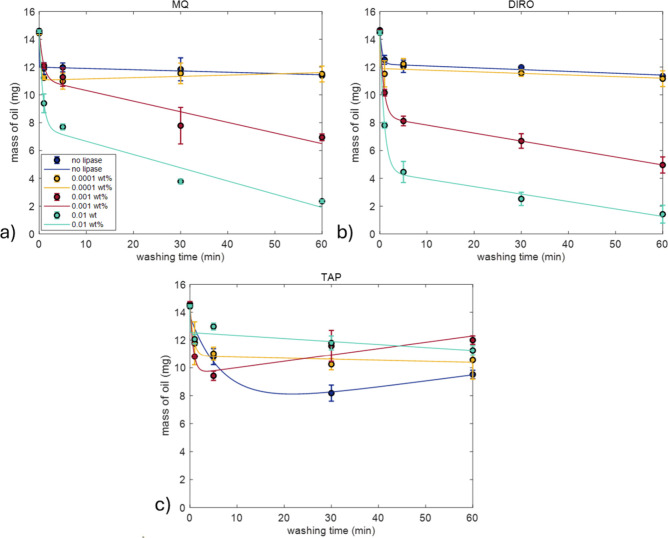
Single-cycle washing experiments. Average amount of olive oil remaining on the surface of plastic tubes (mg) after washing at 25 °C for 1, 5, 30, and 60 min using (a) MQ, (b) DIRO, and (c) TAP. Results are shown for conditions without lipase (blue) and with lipase (yellow, red, green) dissolved in the respective water grades. Each condition was tested in triplicate. Fitting parameters can be found in Table S3. Data is fitted with eq S1. The legend is the same for all panels.

Although different water grades can remove part of the oil from the plastic surface, the surface’s hydrophobic nature and the nonpolar characteristics of both the oil and plastic make the oil removal challenging.

The addition of lipase to purified water grades significantly enhanced their washing efficiency. As the lipase concentration increased, the washing efficiency increased as well. At low lipase concentrations (0.0001 wt %), the effect was minimal when compared to washing with no lipase. However, once the concentration increased above 0.001 wt %, the efficiency greatly increased both for MQ and DIRO. Regarding washing time, the 30 and 60 min wash had the higher efficiencies. After 30 min of washing with 0.01 wt % RNL, washing efficiency reached 74.2% for MQ and 82.6% for DIRO and washing for 60 min further improved these values to 83.7 and 90.2%, respectively. Overall, at 60 min, adding 0.01 wt % lipase increased washing efficiency by 75% relative to the enzyme-free control in both MQ and DIRO.

Importantly, a time dependence was observed only in the presence of lipase. In the enzyme-free experiments, washing efficiency did not exhibit an increase with time, suggesting that without lipase, the interfacial conditions between water and oil remain largely unchanged during washing. By contrast, when lipase was added to the system, the enzymatic activity continuously acted at the oil/water interface, creating a time-dependent trend in washing efficiency. This suggests that the effect of lipase is not instantaneous but accumulates over time as the enzyme catalyzes the olive oil lipids at the interface.

In the case of using TAP with lipase, the washing efficiency showed the opposite trend since it decreased when compared to washing with no lipase. Regardless of the washing duration, the addition of lipase did not promote oil removal.

The addition of olive oil into a solution containing 0.1 wt % lipase in TAP water generated birefringent domains under cross-polarized light conditions, as seen in Figure 6c,d. This figure displays brightly colored textures characteristic of liquid crystalline anisotropic phase ordering.^32^ The likely origin of this structure is the enzymatic hydrolysis of olive oil triglycerides by lipase, resulting in the formation of amphiphilic molecules, such as free fatty acids and monoacylglycerols. These products are capable of self-assembling in aqueous environments into ordered mesophases,^33^ especially under the influence of ionic conditions provided by the SP buffer.^34–36^ Different ions may contribute to phase stabilization by screening electrostatic interactions of the head groups and by letting the tails pack more tightly.^37^ In the case of TAP water, the calcium ions that are present may also promote aggregation by binding to fatty acid head groups, thereby reducing their solubility and helping the precipitation into ordered phases.^38^ The observed increase in residue formation with higher lipase concentrations suggests that the extent of triglyceride hydrolysis and thus the concentration of amphiphiles play a key role in driving the phase transition. The resulting structures may be hexagonal liquid crystalline phases, which are typical for systems rich in fatty acid-based amphiphiles. Overall, the evidence points toward a reaction-driven self-assembly process where lipase activity generates the building blocks that spontaneously organize into liquid-crystalline structures.

**Figure 6:**
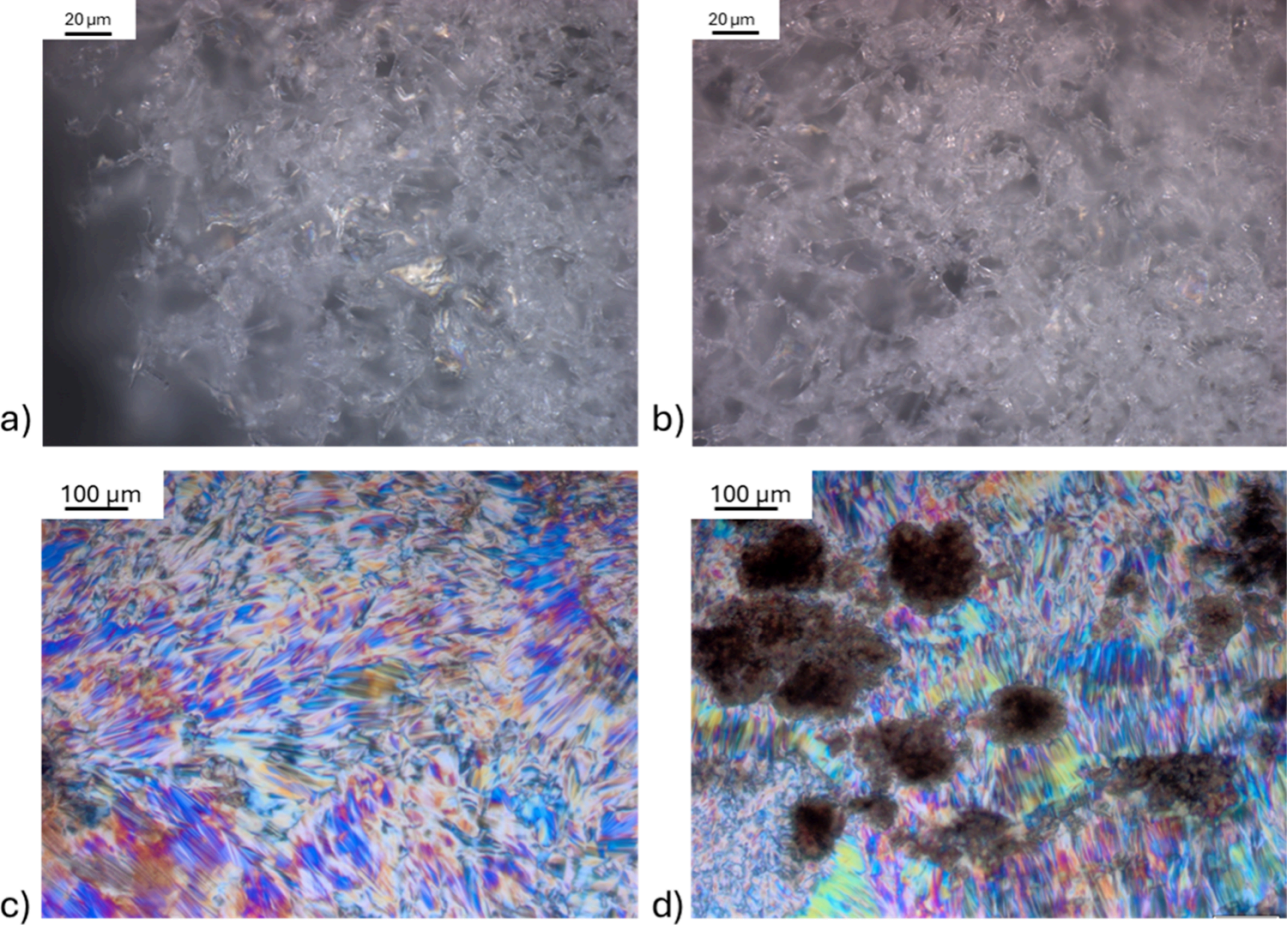
Cross-polarized light microscopy images from two separate experiments. (a, b) Residues formed after washing olive oil with 0.0001 wt % lipase in (a) TAP water and (b) 0.1 M SP buffer. (c, d) Liquid crystal phases obtained in a separate experiment after adding 7–8 mg olive oil to 0.1 wt % lipase solutions in (c) TAP water and (d) 0.1 M SP buffer.

Because the 0.025 M SP buffer appeared to prevent observable residues of liquid crystals within 30 min, all further experiments were standardized to a 30 min wash and SP concentration of 0.025 M, unless otherwise stated.

### 3.3 Effect of Number of Washing Cycles and Temperature

Further research focused on the influence of the washing cycles and temperature on washing plastic surfaces with lipases. The amount of residual oil was measured after washing for 4 cycles, each cycle lasting for 30 min, at 25 and 40 °C.

For 25 °C, purified water grades show increased efficiency with increased lipase concentration (Figure 7a,b). In MQ water, 0.001 wt % lipase required four cycles to reach 97% efficiency while 0.01 wt % lipase removed all surface oil after just two washing cycles. For DIRO water, addition of 0.001 wt % lipase had a 94.7% efficiency while increasing the concentration to 0.01% increased efficiency to 97%. The lowest concentration, 0.0001 wt %, was able to remove more oil than the enzyme-free system, but its efficiency was not enough to fully remove the olive oil from the surface.

**Figure 7:**
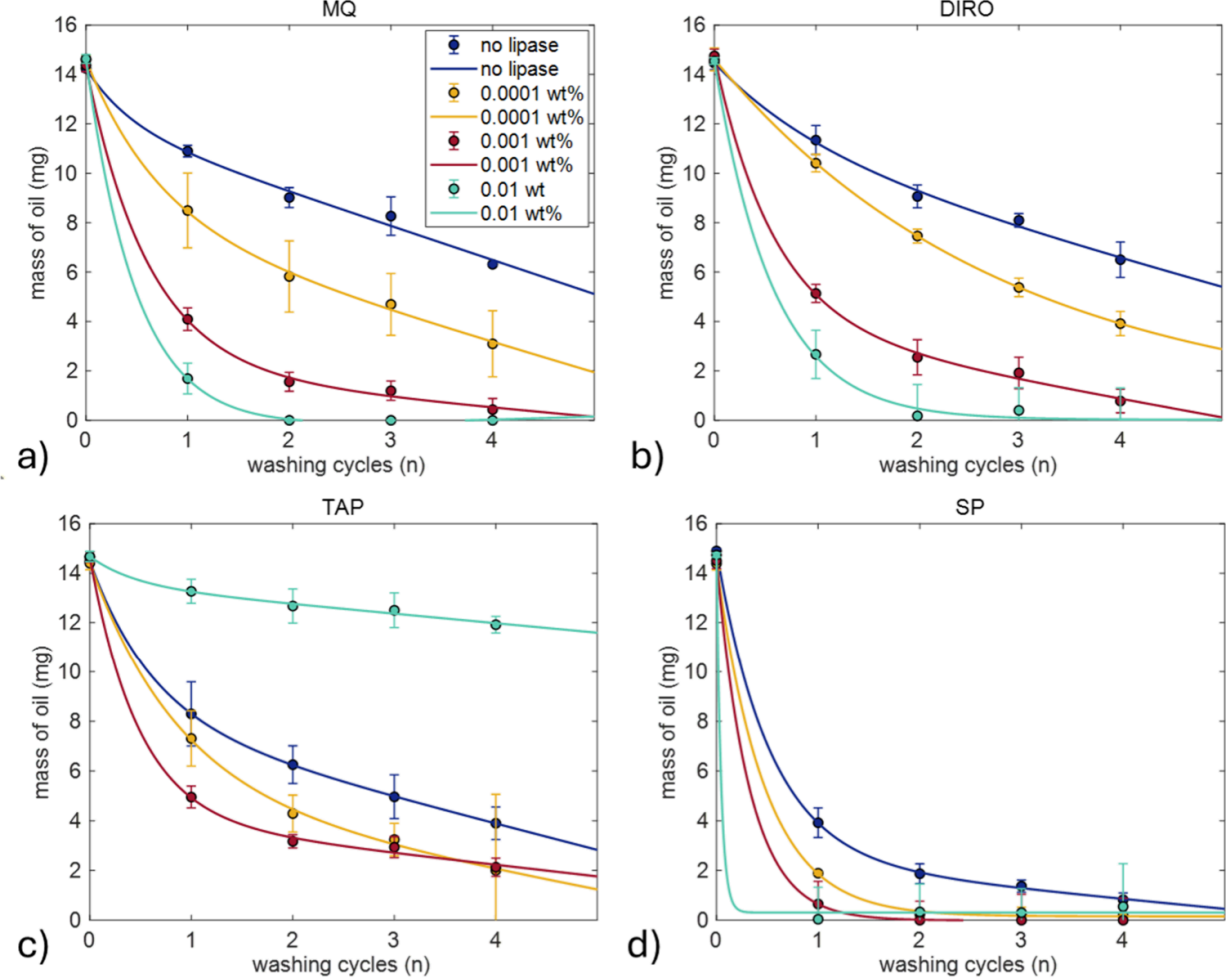
Average amount of olive oil remaining on the surface of plastic tubes (mg) after washing at 25 °C for 1, 2, 3, and 4 washing cycles using (a) MQ, (b) DIRO, (c) TAP, and (d) SP 0.025 M. Each cycle lasts 30 min. Results are shown for conditions without lipase (blue) and with lipase (red, yellow, green) dissolved in the respective grades. Each condition was tested in triplicate. Fitting parameters can be found in Table S2. The legend is the same for all panels.

When TAP water was used, the washing efficiency increased with lipase concentrations of 0.0001 and 0.001 wt %, but for 0.01 wt %, the pellet formation was excessive, resulting in almost no decrease of the mass (Figure 7c). SP data shows that after two washing cycles, the oil is more than 90% removed from the surface irrespective of the lipase concentration (Figure 7d).

At 40 °C, the washing efficiency trend is similar to the one found at 25 °C. As the lipase concentration increases, the washing efficiency increases as well (Figure 8). For purified water grades, addition of 0.0001 wt % lipase had almost the same effect as before. At 0.001 wt %, the washing efficiency reached 95 and 83% for MQ and DIRO, respectively (Figure 8a,b). For the highest concentration, complete oil removal was achieved for both MQ and DIRO after the third washing cycle. TAP water again had a decrease in washing efficiency with the highest lipase concentration, probably due to the formation of liquid crystal residues (Figure 8c). For SP, data show that again after two washing cycles, the oil is more than 90% removed from the surface (Figure 8d).

**Figure 8:**
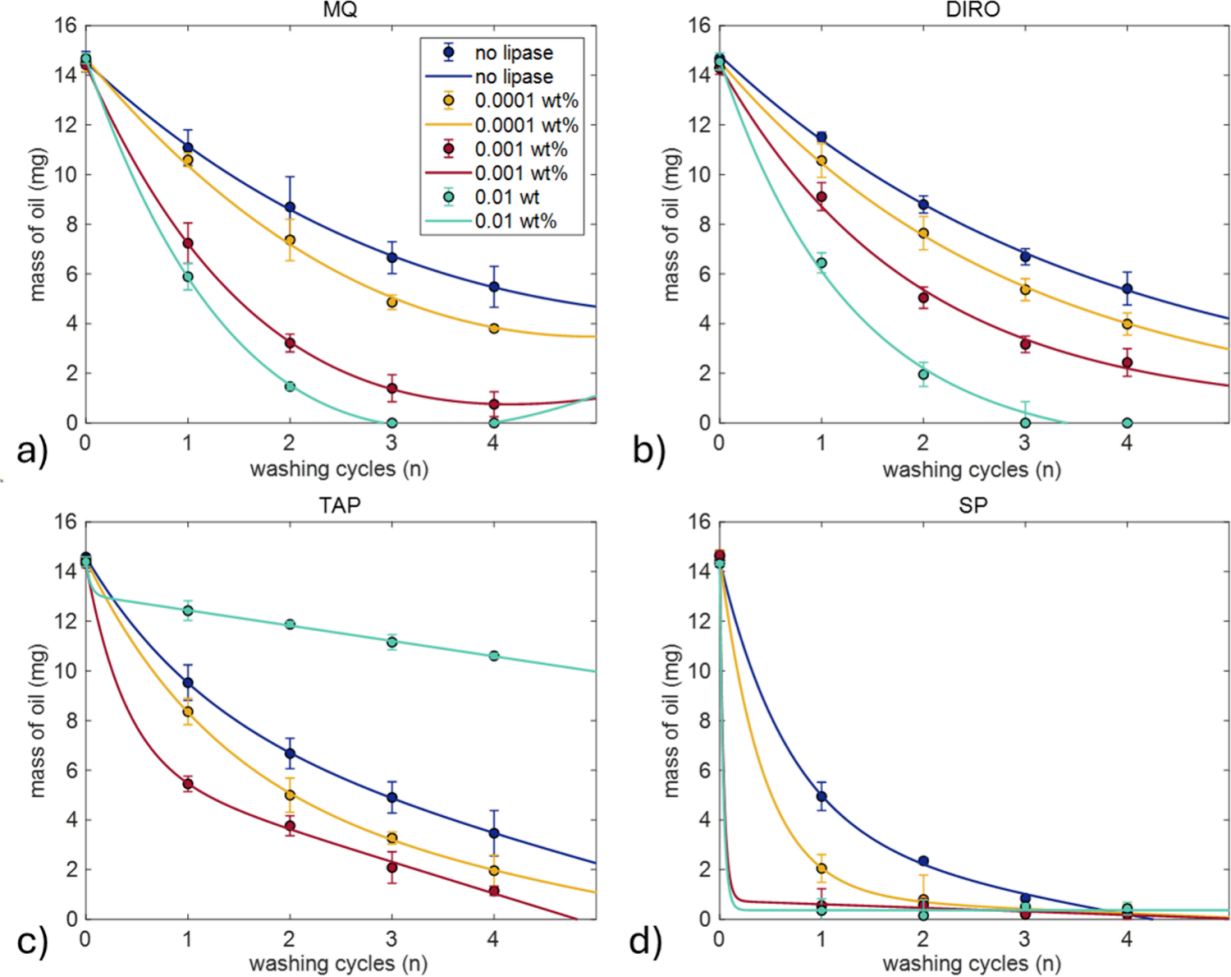
Average amount of olive oil remaining on the surface of plastic tubes (mg) after washing at 40 °C for 1, 2, 3, and 4 washing cycles using (a) MQ, (b) DIRO, (c) TAP, and (d) SP 0.025 M. Each cycle lasts 30 min. Results are shown for conditions without lipase (blue) and with lipase (red, yellow, green) dissolved in the respective grades. Each condition was tested in triplicate. Fitting parameters can be found in Table S2. The legend is the same for all panels.

The effect of the temperature can be better understood through Figure S3. Raising the washing temperature from 25 to 40 °C results in small changes in the oil removal. When MQ water was used (Figure S3a,b), washing at 25 °C removed slightly more oil than at 40 °C during the first two cycles. However, by the third cycle, the cleaning efficiency at both temperatures changed, resulting in comparable levels of oil removal. When TAP water was used (Figure S3c,d), washing for 4 cycles at both temperatures results in almost the same efficiency in all different cycles. The effect of all mentioned parameters on the oil removal is summarized in Table S4.

To understand why lipase is able to remove oil from the surface, the absorption of lipase on the surface and its enzymatic reaction should be understood. In this work, the fungal lipase from *Rhizopus niveus* was the enzyme in use (Figure 1). RNL is a 120 kDa carboxylic-ester hydrolase that cleaves ester bonds in triglycerides.^27,39^

As stated above, at pH values above 3, the lipase carries a net negative charge, similar to that of polypropylene plastic surfaces in aqueous environments.^40,41^ This suggests that in theory, electrostatic repulsion could occur, preventing adsorption. However, in practice, enzymes can orient themselves to expose locally positive surface patches, enabling adsorption despite their overall negative charge.^42^ This indicates that hydrophobic interactions rather than electrostatic forces are the main driving factor for adsorption. Once the lipase comes into contact with the olive oil–coated plastic surface, the degradation process can begin.

RNL hydrolyzes triglycerides at its active site, which consists of a catalytic triad of serine, histidine, and aspartic or glutamic acid residues, with specificity for the sn-1 and sn-3 positions.^19^ The reaction proceeds in a stepwise manner, generating various intermediate products, including diglycerides (eq 1) and monoglycerides (eq 2), before ultimately yielding free fatty acids and glycerol (eq 3).^43^

$${\left(\mathrm{RCOO}\right)}_{3}C_{3}H_{5}+H_{2}O\overset{\mathrm{lipase}}{\to }{\left(\mathrm{RCOO}\right)}_{2}C_{3}H_{5}\left(\mathrm{OH}\right)+\mathrm{RCOOH}$$ 1

$${\left(\mathrm{RCOO}\right)}_{2}C_{3}H_{5}\left(\mathrm{OH}\right)+H_{2}O\overset{\mathrm{lipase}}{\to }\mathrm{RCOO}-C_{3}H_{5}{\left(\mathrm{OH}\right)}_{2}+\mathrm{RCOOH}$$ 2

$$\mathrm{RCOO}-C_{3}H_{5}{\left(\mathrm{OH}\right)}_{2}+H_{2}O\overset{\mathrm{lipase}}{\to }C_{3}H_{5}{\left(\mathrm{OH}\right)}_{3}+\mathrm{RCOOH}$$ 3

A movable amphipathic “lid” shields the catalytic pocket. In the inactive, closed state, the lid presents a hydrophilic face to the surrounding solvent; upon interfacial binding, the lid opens, exposing the hydrophobic residues of the active site and permitting substrate hydrolysis.^24^ Because of its hydrophobic site, the lid will open and activate the enzyme only when it is in contact with a hydrophobic material.

Lipase activation is not exclusively triggered by triglycerides but by exposure to any sufficiently hydrophobic interface.^44–46^ Contact with a polypropylene plastic surface can therefore induce lid opening, as the hydrophobic polymer stabilizes the otherwise energetically unfavorable exposure of apolar residues near the active site. In this state, the enzyme can adsorb onto the plastic, even in the absence of lipid substrates. However, when a triglyceride ‘rich’ phase such as olive oil is introduced onto the same surface, competitive adsorption processes dominate. The oil/water interface provides a stronger hydrophobic driving force and a more favorable environment for orienting the lipase.^47,48^ As a result, lipase molecules already adsorbed on plastic are likely to undergo partial desorption and redistribute to the oil–water interface, where their catalytic efficiency is maximized. This dynamic equilibrium reflects the relative strengths of hydrophobic stabilization that shows that the adsorption to hydrophobic solids can open the lid, but the presence of a natural substrate interface generally outcompetes the inert plastic, leading to enhanced localization and activity of lipase in the oil phase.

Once the enzyme is in contact with the oil, its products will be formed. Glycerol, which is the final product of this catalysis, is fully soluble. As soon as it is formed, it will directly dissolve into the aqueous phase.^49^ Free fatty acids (RCOOH) are produced after each step and can be easily deprotonated in the presence of hydroxide ions. This process will lead to the formation of surface active carboxylate ions (RCOO^–^) and water (eq 4).

$$\mathrm{RCOOH}\;(\mathrm{fatty}\;\mathrm{acid})+{\mathrm{OH}}^{-}⇌\mathrm{RCO}O^{-}+H_{2}O$$ 4

The negatively charged carboxylate group enables the fatty acid to interact with polar molecules such as water. This characteristic makes the anionic fatty acids amphiphilic, surface active, and easier to be removed from the surface. The diglycerides and monoglycerides produced by these reactions are used as emulsifying agents. Due to their amphiphilic nature (hydrophilic tail and hydrophobic head), which allows them to adsorb at the oil/water interface, they can reduce the interfacial tension and stabilize dispersed oil droplets in the aqueous phase.^50^ This prevents recoalescence or readsorption of oil onto the surface, facilitating effective removal during washing.

With the mechanism of lipase adsorption and hydrolysis understood and established, we now look into how lipase addition into different water grades can improve the washing of hydrophobic surfaces from hydrophobic oily soils.

Buffer solutions exhibit high efficiency, irrespective of the presence of enzyme. At an almost neutral pH (7.8 as shown in Table S1) free fatty acids that are always present in edible oils^51^ are deprotonated to their anionic forms. Those anionic fatty acids migrate to the oil–water interface and decrease the interfacial tension. They are acting as surfactants, stabilizing oil droplets in water and preventing oil films from adhering to the plastic surface.^52^ Moreover, studies have shown that even at low concentrations (less than 10 mM), phosphate buffer reorganizes triolein (used as a model compound for triglycerides, which is also found in olive oil^51^), reducing its interfacial tension.^53^ This decrease in the surface tension makes it easier for the oil to be dispersed in water and washed away. In contrast, this process does not occur as easily in pure water, where oil tends to adsorb onto the surface rather than dissolve in it.

When lipase is added into the water and the SP buffers, the efficiency is increased compared to the lipase-free washing. Figure 7 shows that SP can remove oil faster from the surface, compared to purified water grades. The adsorption and catalytic efficiency of RNL on olive oil-coated plastic surfaces are expected to depend strongly on the ionic strength of the surrounding medium. In MQ water, where ionic strength is low, electrostatic repulsion between the negatively charged enzyme and anionic species at the interface is unscreened, leading to slower adsorption and reduced initial hydrolysis rates. Nevertheless, hydrophobic interactions still drive lid opening and binding, so catalysis proceeds, although slower. In TAP water, the presence of ions facilitates the adsorptions and the higher hydrolysis rates, but addition of high lipase concentration can promote the formation of pellets. At low phosphate concentrations (0.025 M), electrostatic repulsion is partially screened, allowing faster and more stable adsorption of lipase at the oil/water interface. These conditions favor the activation of lipase and promote the formation of negatively charged carboxylate groups, diglycerides, and monoglycerides, resulting in a faster oil removal. Addition of higher phosphate concentration (0.1 M) might screen even more the electrostatics, but the extensive generation of amphiphilic hydrolysis products led to the spontaneous appearance of birefringent liquid crystalline phases, making the use of high buffer and lipase concentration impractical.

The adsorption and enzymatic reactions are processes that require time. The slower adsorption observed in MQ water can be compensated by increasing the lipase concentration. While a single washing cycle may not completely remove the olive oil, higher enzyme levels are sufficient to achieve full hydrolysis of the surface-bound oil in only 2 cycles.

Although the lipases optimal working temperature is 40 °C,^54^ the washing efficiency did not improve when this temperature was used. On the contrary, for the purified water grades, the washing efficiency exhibited a small decrease. A possible explanation for that would be the inhibition of the enzyme from the hydrolysis products. The long-chain free fatty acids form tight complexes with lipases. As soon as enough product has accumulated, a fraction of the enzyme is locked up in these complexes, removing it from action.^55,56^ Moreover, the di- and monoglycerides that are formed can be deposited on the oil/water surface and ‘block’ the enzymatic activity by preventing it to interact with the oil.^57^

To detect if lipases can be used for removing lipids from different surfaces, such as hydrophilic surfaces, QCM-D was used.

### 3.4 Effect of Lipase on Glycerol Dioleate Removal from Solid Surfaces: QCM-D Study

The QCM-D method was used to investigate how the RNL lipase can remove lipids from a hydrophilic silica surface. Glycerol dioleate (GDO) was used as a model lipid since it has a higher purity than olive oil, which is important for the sensitive QCM-D method. Under the experimental conditions used, GDO formed liquid droplets on the sensor surface rather than forming a thin film (Figure S7). The silica sensors were coated with GDO and were washed with either buffer or aqueous solutions with or without lipase for 1 h. The experiments consisted of four regimes: A – bare sensor in air, B – sensor coated with GDO in air, C – sensor with GDO in water, and D – sensor after treatment with water and drying (Figure 9, Figures S4–S6). The frequency and dissipation measurements in air were performed in five replicates to minimize the error arising from remounting the QCM-D sensor.

**Figure 9:**
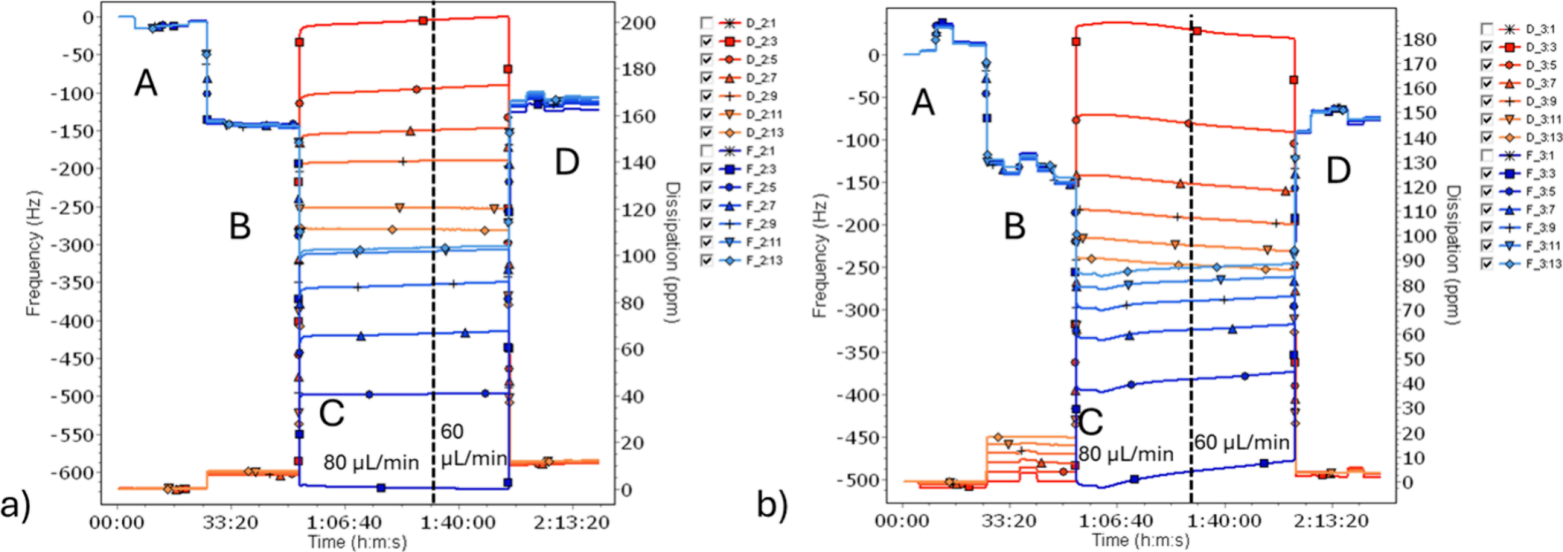
QCM-D results for MQ water without lipase (a) and MQ water with lipase (b). Frequency (blue) and dissipation (red) of overtone 3:11 are shown. The four regimes are A, bare sensor in air; B, sensor coated with GDO in air; C, sensor with GDO in liquid; D, dried sensor after treatment with liquid. Experiments in air were repeated 5 times, and repetitions are shown combined in regimes B and D. The film thicknesses in the B and D regimes are calculated by the Voigt model.

When the medium changes from air to liquid and vice versa (from regimes B to C and from C to D), stepwise shifts of frequency and dissipation are clearly seen. In cases in which water was used for the washing, the frequency and dissipation values were relatively stable during the washing part. When lipase was present, the results varied depending on the water grade used. When purified water grades were used, both frequency and dissipation showed a trend (Figure 9, Figure S5), where frequency was increasing, i.e., Δ*f* becoming less negative and dissipation slightly decreasing. When TAP water was used, no trends were observed (Figure S5). When sodium phosphate buffer was used, a trend was seen, irrespective of the use of lipase (Figure S6).

Calculations of effective film thickness from the frequency and dissipation data presented a challenge since the lipid did not form a smooth film on the surface; see microscopy images in Figure S7. Hence, the Sauerbrey model was deemed unsuitable since it assumes a uniform elastic thin film coating the surface. Although QCM-D theory is best developed for the cases of smooth films, there are research works showing that liquid droplets can also be studied using this method.^58,59^ In the case of droplets, the expected response is viscoelastic^60^; therefore, we applied the Voigt model for calculating the effective thickness of GDO film in air. Moreover, this approach was also useful in analyzing regime C (washing step) where the system was exposed to the aqueous liquid. By fitting the lipase-washing regime to the Voigt model, we could better understand how the enzyme acted on the surface and whether its efficiency was dependent on the properties of the aqueous phase.

The amount of GDO removed from the surface was calculated by the difference in the film thickness between regimes B and D. Hence, it characterizes the amount of lipid removed not only during the washing regime but also during the regime change. Table S6 shows the calculated values of the thickness when the Voigt model was used. The system with lipase was modeled as consisted of a viscoelastic film (consisting of two layers, layer 1 corresponds to GDO and layer 2 corresponds to RNL), described using the Voigt model in a semi-infinite fluid of viscosity of 0.89 mPa·s for all grades of water and buffer. The densities of GDO, water, and RNL (0.934,^61^ 0.997, and 1.350 g/cm^3^, respectively) were kept constant, while viscosity, shear modulus, and thickness of the film were used as fitting parameters. The thickness of both layers was allowed to vary with time. Figure 10 shows how the combined thickness changes over time. In such a complex system where lipase acts on liquid oil droplets and liquid crystalline phases can form, combining the thicknesses provides a clearer and more reliable overall representation. The full set of the fitting parameters is shown in Table S5.

**Figure 10:**
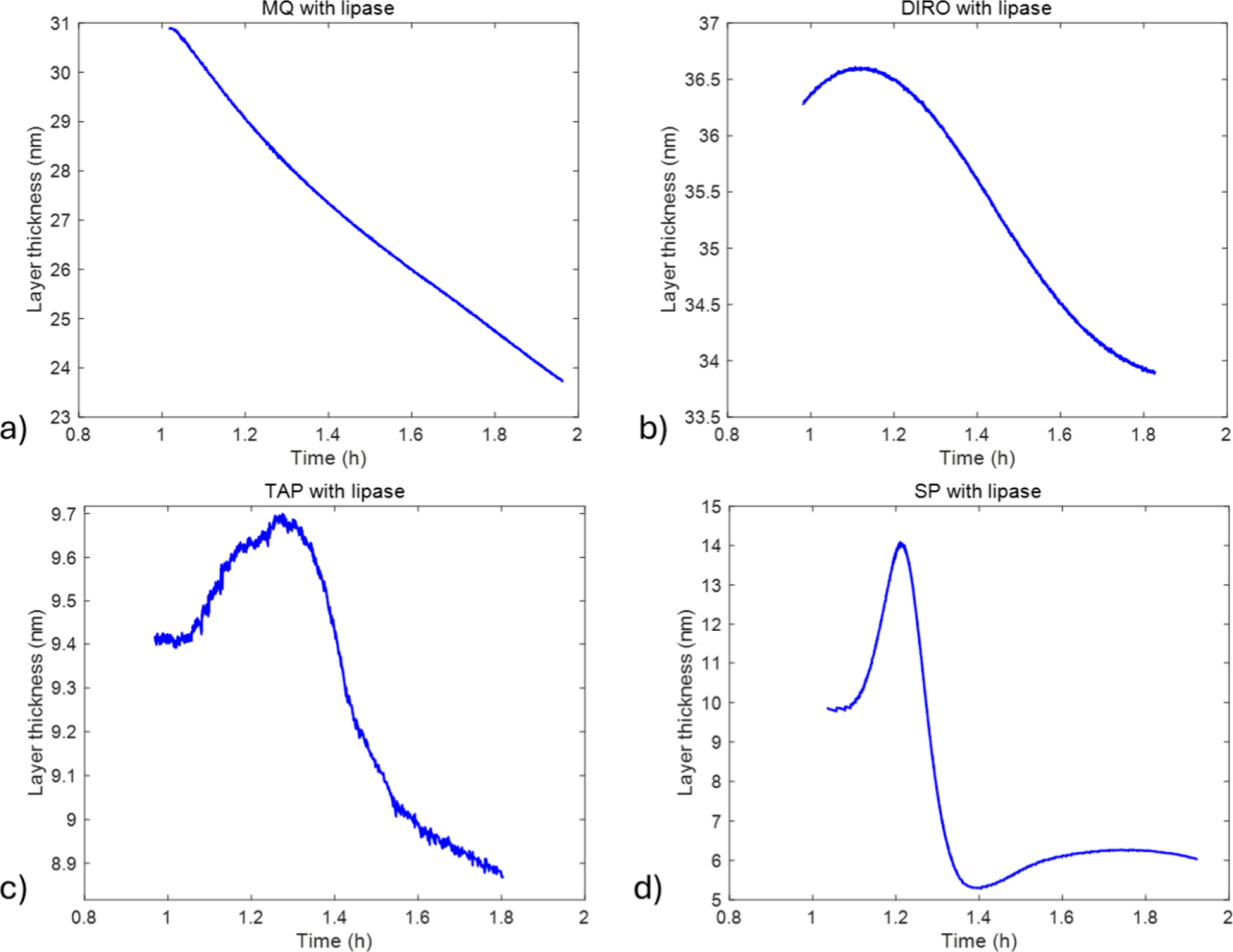
Combined thicknesses of GDO and RNL layers during washing experiments with (a) MQ, (B) DIRO, (c) TAP, and (d) sodium phosphate 0.025 M buffer.

In the initial part of the washing with DIRO, TAP, and SP, an increase in thickness is observed (Figure 10). MQ water did not follow the same trend. After that initial step, in all cases, the layer has a decreasing trend. To fully understand what is happening in the system, we should describe the process from the beginning. It is known that GDO forms reverse micellar structures^61^ both in the presence and absence of water. Upon contact with water, the reverse micellar structures are expected to take up a certain amount of water molecules in their cores. In addition to reverse micelles, the GDO–water interface may exhibit an altered structure in which the lipid molecules orient their head groups toward the water phase. Once lipase is introduced into the system, it can begin to adsorb onto the GDO layer, which may be observed as an initial increase in the film thickness for the DIRO, TAP, and SP samples.

In bulk water, lipase is in its ‘closed’ conformation and will show little to no activity. When the lipase reaches the GDO, its hydrophobic regions will bind the lipase, and that will trigger the ‘lid’ of the enzyme to open. Opening the lid both anchors the enzyme to the oil GDO droplets (hydrophobic patch into lipid, polar region toward water) and switches it to a catalytically active state. Once the lipase is in contact with GDO, the catalytic activity will begin and GDO will degrade into monoolein, oleic acid, and glycerol:

$$\mathrm{GDO}+2H_{2}O\overset{\mathrm{lipase}}{\to }\mathrm{Glycerol}+2\mathrm{Oleic}\;\mathrm{acid}$$ 5

As oleic acid, monoolein (intermediate product), and glycerol are formed, the mass adsorbed on the surface continuously changes as these products reorganize or are removed from the surface. This removal of the products is translated into the increase of the frequency during the washing regime (Figures S4–S6) and the fast decrease on the mass of the layer shown in Figure 10.

The above experiment indicates that indeed, GDO can be removed from the surface; however, calculating the precise efficiency remains challenging, as the thickness of the layer after washing (Table S6) is different than the actual layer that is present on the surface. The difference in the mass could be derived either from bound water or by the precipitation of ions on the surface since no rinsing was conducted.

Overall, the QCM-D results demonstrate that water alone can remove part of the GDO layer but this washing is less effective than washing with lipase. When purified water is used alone, soil removal occurs mainly at the beginning of the washing process during the transition from air to water. The addition of lipase significantly enhances lipid removal through enzymatic hydrolysis; however, a substantial portion of the oil remains on the surface after the reaction. The conditions required for the complete removal of this residual layer warrant further investigation.

To further understand and investigate the mechanisms of removal of oil from surfaces by water grades and lipases, we performed contact angle measurements in hydrophilic and hydrophobic surfaces.

### 3.5 Effect of Lipase on Wetting and Spreading Behavior of Oil at the Interfaces

To detect whether the presence of lipase in water or buffer solutions changes the wetting behavior of olive oil, experiments were performed using a glass surface. In these tests, aqueous solutions with or without lipase were added to the glass surface containing an olive oil droplet. The underwater contact angle was measured for 10 min.

Figure 11 shows images of oil droplets under MQ water or lipase solutions almost directly after the addition of the aqueous liquid. The droplets in the bottom part of the images are very small in all cases, showing that a large part of olive oil detached during addition of water or lipase solution. This observation once again highlights importance of phase change (from air to water), which further facilitates the cleaning process. After the initial stage, the droplets on the solid surface did not change over time, which is clear from the stable values of the contact angle (Figure 11a). In the experiments where 0.01 and 0.1 wt % of lipase was used, there was a bigger droplet formed at the air/water interface. This behavior was absent in the first two images, which correspond to samples with no lipase and 0.001 wt % lipase. In the cases when the droplets were seen, after a certain time, they disappeared from the view (see also Video S1), probably moving to the hydrophobic walls of the cuvette.

**Figure 11:**
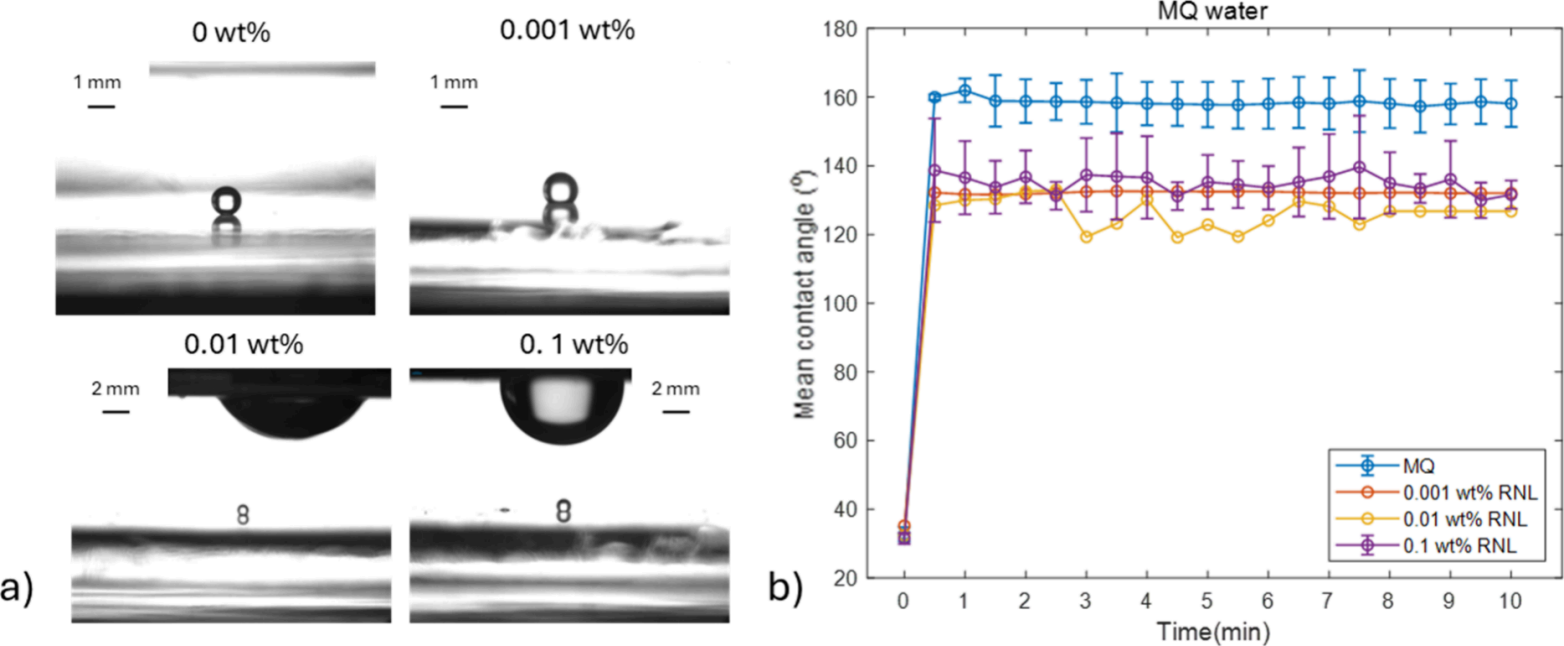
Contact angles of olive oil in MQ water with and without addition of lipase on the glass surface. (a) Representative frames show oil droplets with 0, 0.001, 0.01, and 0.1 wt % lipase concentrations after addition of the liquid phase. (b) Mean contact angle (±SD) of olive oil (°) as a function of time (min). The initial contact angle of oil in air is shown at time zero, and then measurements in water were performed every 30 s for 10 min, using triplicate samples. Contact angles were determined via the sessile drop technique, and the Young–Laplace equation was used to estimate the angles.

This phenomenon was also present in experiments with TAP water, SP of 0.05 and 0.1 M (Figures S9–S11). In the presence of TAP water, the upper droplets were again found when 0.01 and 0.1 wt % of lipase were used. When SP was used with 0.001 and 0.01 wt % of lipase, the oil formed either one big oil droplet or many small ones on the water/air interface. In all cases, the droplets were formed only when lipase was used in the system, and typically the contact angle increased with increasing lipase concentration (see Table S7).

The contact angle of the lower oil drop in water was observed over a period of 10 min to detect whether the lipase would promote a change in the angle. When MQ water was used with no addition of lipase, the contact angle remains steady at approximately 160° over a period of 10 min (Figure 11b). When lipase was present in the system, the contact angle was lower but no significant changes were observed over time. When TAP water was used, the contact angle was lower in experiments with lipase, while when buffers were used, the presence of lipase did not change the contact angle. In all cases, no time dependence was detected (Figures S9–S11).

The contact angle of the oil droplet at the air/water interface for the case of 0.1 wt % lipase in MQ water was measured to be 96.3°. To understand why the upper drop is formed only in the presence of lipase, the spreading behavior of the oil must be considered. When a small volume of a liquid is placed on the surface of another immiscible liquid, it can either spread spontaneously to form a thin film or remain as a droplet with a defined contact angle. The outcome depends on the balance of the interfacial tensions at the three-phase contact line (air, water, oil).^62,63^

The spreading behavior is quantitatively described by the spreading coefficient (*S*), defined by eq 6:^62,63^

$$S=\gamma_{\mathrm{wa}}-\left(\gamma_{\mathrm{wo}}+\gamma_{\mathrm{oa}}\right)$$ 6

where γ_wa_ is the interfacial tension between water and air, γ_wo_ is the interfacial tension between water and oil, and γ_oa_ is the interfacial tension between oil and air. A positive spreading coefficient indicates that the oil spreads spontaneously on water, forming a thin film; a negative value means that it stays as a discrete droplet at the water/air interface. While the spreading coefficient provides a criterion for spreading, the contact angle of the formed droplets can be further understood through Young’s equation, which relates the interfacial tensions to the observed droplet.

$$\mathrm{cos\theta}=\frac{\left(\gamma_{\mathrm{wa}}-\gamma_{\mathrm{oa}}\right)}{\gamma_{\mathrm{wo}}}$$ 7

To calculate the spreading coefficient and the contact angle, the surface and interfacial tensions of the systems were measured (Figures S12–S15) and presented in Table 1. By using the experimental interfacial tension values in eqs 6 and 7, the spreading coefficient and the contact angle at the air/water interface can be quantitatively evaluated for both the lipase and no-lipase systems.

**Table 1:** Mean Experimental Results from γ_wo_, γ_wa_, and γ_oa_ for the Olive Oil in Water, in 0.1 wt % Lipase in MQ Water, and for the Oil with 0.1 wt % Lipase in MQ water.^b^

	water	0.1 wt % lipase^a^	oil with 0.1 wt % lipase
γ_ow_	20.0	17.0	
γ_wa_	69.2	63.0	28.1
γ_oa_	30.15		
*S*_cal_	19.0	15.8	–19.0
cos(θ)_cal_	1.9	1.9	–0.1
θ_cal_	0	0	96.8
θ_exp_	0		96.3

a This case considers equilibrium between the three phases but assumes no effect of oil on the surface tension of lipase solution γ_wa_.

b *S*_cal_, cos­(θ)_cal_, and θ_cal_ are the calculated spreading coefficient, cosine of the contact angle, and the contact angle, respectively. Examples of the interfacial tension measurements are shown in Figures S12–S15.

In the system without lipase, substituting the values into eq 6 results in a spreading coefficient of 19 mN/m. Since *S* is positive, the oil phase will spontaneously spread at the water/air interface, forming a film, confirming that spreading is the thermodynamically favored outcome at the system without lipase. In the experiments with lipase solution, the oil/water interfacial tension reduces to 17 mN/m and the water/air surface tension to 63 mN/m. These changes would result in a positive spreading coefficient of 15.8 mN/m, corresponding to spreading of oil and the zero contact angle (θ). However, while in the case of pure water, the spreading is indeed observed, in the case of 0.1 wt % lipase solution, the oil forms a distinct droplet with a relatively high contact angle.

To understand the reason for this discrepancy, it is necessary to examine the case in which olive oil is explicitly present during the measurement of air/water surface tension γ_wa_. For this experiment, a mixture of olive oil and lipase-containing water were used, as in the contact angle measurements. The mixture was briefly vortexed and then allowed to equilibrate for 2 h. In that case, when both lipase and dispersed oil were present in the aqueous phase, the air/water surface tension was measured to be 28.1 mN/m. This value is far lower than the one obtained in the absence of olive oil (γ_wa_ = 63 mN/m in the absence of oil), indicating that the presence of oil molecules together with lipase at the water/air interface strongly influences both the spreading coefficient and the contact angle. The calculated spreading coefficient is −19.0 mN/m (suggesting formation of a droplet at the air/water interface), while the calculated contact angle is 96.8°. In agreement with this, the experimentally measured contact angle of the droplet was 96.3°, closely matching the theoretical prediction.

From the experiment, it is seen that the interaction between the olive oil and the lipase is vital for the decrease of the surface tension and the formation of the droplet. The decrease of the surface tension is evidence that lipase hydrolyzes the triglycerides present in the olive oil. The hydrolysis results in the formation of amphiphilic free fatty acids (FFA). When in water, some FFAs could migrate toward the air/water interface and others could disperse into water and start forming micelles. The FFAs that adsorb at the interface can reduce the surface tension, acting like surfactants. This decrease in the surface tension signifies that less energy is required for the formation of the air/water interface; thus, extending it at the expense of other interfaces is thermodynamically favorable.

The hydrolysis of triglycerides accelerated by lipase takes place both in water and in buffer solutions. Gravimetric analysis indicates that, in phosphate buffer, the increased ionic strength screens electrostatic repulsions, allowing lipase to adsorb more readily onto the olive oil surface and further affecting the interfacial tension.

In brief, the results demonstrate that the addition of lipase to purified water significantly enhances the removal of thick olive oil layers from hydrophilic and hydrophobic surfaces. Gravimetric analysis confirms that the presence of small amounts of lipase in purified water is sufficient to achieve nearly full removal of olive oil from hydrophobic surfaces. To understand the mechanism behind this efficient cleaning action, it is necessary to consider interfacial tension experiments. In this system, when water with lipase is added to an olive oil droplet on a hydrophilic surface, most of the oil is detached and forms a separate droplet at the air/water interface. Although air is not hydrophobic in the strict chemical sense, the air/water interface behaves as a hydrophobic boundary because it is nonpolar and unfavorable for hydrogen bonding. Hence, the water/air interface can serve as a model interface between hydrophilic and hydrophobic phases.

This interfacial environment promotes the adsorption of FFAs at the interface. Consequently, while the hydrophobic olive oil tends to spread over a hydrophobic interface, the interfacial forces and the action of lipase promote the formation of a droplet rather than a continuous film. Notably, while the influence of lipase on the contact angle at a hydrophilic interface is not pronounced, it strongly increases the contact angle of oil at the hydrophobic interface. The droplet could then be more easily detached or removed through gentle mechanical agitation, as observed in the gravimetric experiments. Overall, these findings highlight the role of lipase in promoting the efficient removal of hydrophobic soils by using purified water grades.

Lipase addition to water represents an ecofriendly and sustainable alternative to traditional detergent-based cleaning, combining biodegradability and energy efficiency with effective fat removal. Although we cannot claim that purified alone can completely clean the hydrophobic surface, traces of lipase can be added to fully remove the olive oil from the surfaces.

## 4 Conclusions

This study demonstrates that low concentrations of lipase in purified water or buffer solution can effectively remove hydrophobic soils, such as olive oil and glycerol dioleate (GDO), from both hydrophilic and hydrophobic surfaces. Gravimetric analysis showed that even small amounts of lipase in purified water substantially improved the cleaning efficiency for olive oil at temperatures below 40 °C. Although adding lipase to purified water or buffer markedly enhances washing performance, its addition to tap water does not produce the same improvement.

The enzymatic reaction begins when lipase encounters hydrophobic materials such as olive oil and GDO. Lipase adsorption depends on both hydrophobic interactions and ionic strength. In low-ionic-strength MQ water, electrostatic repulsion slows hydrolysis, whereas low concentrations of phosphate buffer enhance the adsorption and catalytic activity. Increasing the lipase concentration compensates for slower adsorption and enables nearly complete oil removal within two washing cycles. Hydrolysis produces diglycerides, monoglycerides, free fatty acids, and glycerol, all of which are more water-soluble than the original soils. These products not only detach from the surface more easily but also act as natural surfactants that promote roll-up and solubilization. In TAP water, however, the accumulation of surface-active species leads to the formation of liquid-crystalline aggregates, reducing the overall cleaning efficiency.

Contact angle and surface tension measurements show that lipase addition affects interfacial tensions. The strongest decrease occurs at the water/air interface but only in the presence of oil, confirming that the effect is due to hydrolysis products rather than lipase itself. As demonstrated for oil at the water–air interface, these products function as surfactants that shift the balance between interfacial tensions, promoting droplet formation rather than spreading at hydrophobic surfaces and thus facilitating removal.

Overall, lipase enables efficient, surfactant-free cleaning of hydrophobic soils under mild conditions. Further optimization and standardization could support the development of a sustainable enzyme-based “smart” cleaning process that uses purified water for environmentally friendly washing applications.

## Supplementary Material





## Notes

Tables of pH values of the solutions; details of gravimetric analysis; QCM-D data; contact angle measurement data; interfacial tension measurements images (PDF)
Video of oil removal by lipase solution (MP4)

## Abbreviations

*a*: slope of the linear dependence
c: exponential decay constant
GDO: glycerol dioleate
*m°*: initial mass of oil (mg)
*m*: mass of removed oil (mg) after the washing cycle
*m* *_s_ *°**: amount that can be removed during the exponential regime (mg)
*n*: washing cycle number
o: oil
pI: isoelectric point
QCM-D: quartz crystal microbalance and dissipation
RNL: *Rhizopus niveus* lipase
SP: sodium phosphate buffer
*S*: spreading coefficient
γ_wa_: interfacial tension between water and air
γ_wo_: interfacial tension between water and oil
γ_oa_: interfacial tension between oil and air
θ: contact angle
