# Supplementary material for: Removal of Fat from Surfaces by Lipase-Enhanced Purified Water
Source: ACS Omega. 2026 Mar 16;11(12):19391–405. doi: 10.1021/acsomega.5c12931 (PMC13044852; doi:10.1021/acsomega.5c12931)
Supplement: Supplementary file 1 [file ao5c12931_si_001.pdf]

Supporting information

Removal of fat from surfaces by lipase-enhanced purified water

Andriani Tsompou[1,2], Dorina Kokrehel[1,2], Kayleigh Davies[1,2] and Vitaly Kocherbitov[1,2*]

[1] Department of Biomedical Science, Malmö University, SE-20506, Malmö, Sweden

[2] Biofilms research center for Biointerfaces, Malmö University, SE-20506, Malmö, Sweden

*Corresponding author. Department of Biomedical Science, Malmö University, SE-20506, Malmö, Sweden. Phone: +4640-6657946. E-mail: Vitaly.Kocherbitov@mau.se

1. Results

1.1 Conductivity and pH measurements

Table S1: pH of various solutions with and without addition of lipase.

Lipase (wt%)	SP 0.1 M	SP 0.05 M	SP 0.025 M	Milli-Q	DIRO	TAP
0	7.70	7.75	7.81	6.64	7.83	6.68
0.001	7.70	7.75	7.80	6.79	7.68	6.79
0.01	7.70	7.75	7.80	7.52	7.13	6.91
0.1	7.68	7.72	7.74	6.80	6.54	7.18
1	7.55	7.50	7.41	6.53	6.53	6.86

1.2 Gravimetric analysis

All gravimetric data shown in this article is fitted with equation (S1):

$$m = m^o - m_s^o(1 - e^{-cn}) - an \quad \text{(S1)}$$

were m is the mass of removed oil (mg) after the washing cycle, m^o is the initial mass of oil (mg), m_s^o is the amount that can be removed during the exponential regime (mg), c is the exponential decay constant, a is the slope of the linear dependence and n is the washing cycle number. When the washing time is used, equation (S2) is of use, where t is the washing time of the cycle. The equations were used only for plotting the data and its parameters should not be for the washing process. Both equations were used in our previous research [1, 2].

$$m = m^o - m_s^o(1 - e^{-ct}) - at \quad \text{(S2)}$$

Table S2: Calculated values from non-linear fit of gravimetric data obtained from plastic tubes for MQ, DIRO, TAP and SP at 25, and 40°C with and without lipase addition. For the fitting, a lower limit of 0 is used for the linear part (a ≥ 0).

Grade	Temperature (°C)	lipase (wt%)	mo	mos	c	a
MQ	25	0	14.2485	2.2475	2.2679	1.3772

		0.0001	14.5953	6.5537	1.398	1.2208
		0.001	14.3802	12.3767	1.632	0.3739
		0.01	14.6317	14.6989	2.1411	0
	40	0	14.5426	12.5231	0.3226	0.0003
		0.0001	14.5527	15.0828	0.3234	0
		0.001	14.4687	14.783	0.6946	0
		0.01	14.7414	15.5403	0.892	0
DIRO	25	0	14.4732	3.4081	0.9478	1.1375
		0.0001	14.6132	13.8869	0.3567	0.0359
		0.001	14.7566	10.8898	1.7065	0.7496
		0.01	14.5495	14.5259	1.733	0
	40	0	14.7995	14.1701	0.2741	0.0048
		0.0001	14.561	14.1023	0.3433	0.0044
		0.001	14.3895	13.8952	0.5246	0
		0.01	14.6282	15.7353	0.7789	0
TAP	25	0	14.4963	6.3911	1.6276	1.056
		0.0001	14.4139	9.3245	2.0951	0.4665
		0.001	14.6515	10.5584	2.0951	0.4665
		0.01	14.6773	1.1559	2.2664	0.385
	40	0	14.5852	7.0565	0.8352	1.0755
		0.0001	14.4822	10.5514	0.7428	0.6218
		0.001	14.3127	8.185	2.5881	1.2722
		0.01	14.3967	1.335	22.3396	0.618
SP	25	0	14.9008	12.4582	1.9	0.3978
		0.0001	14.3915	14.24	2.1183	0
		0.001	14.5067	14.5165	3.0937	0
		0.01	14.7367	14.4316	22.2902	0
	40	0	14.4395	11.4322	1.4459	0.7129
		0.0001	14.6127	13.7065	2.3367	0.166
		0.001	14.6667	13.9166	26.9774	0.144
		0.01	14.3267	13.9609	29.4934	0

Table S3: Calculated values from non-linear fit of gravimetric data obtained from plastic tubes for MQ, DIRO, TAP and Phosphate buffer at 25 °C with and without lipase addition for washing in different times. For the fitting, a lower limit of 0 is used for the linear part (a ≥ 0).

Grade	lipase (wt%)	Washing time (min)	mo	mos	c	a
Phosphate buffer	0	1	14.4392	3.5784	21.4544	0
	0.0001	5	14.542	6.2269	1.3222	0.0719

(0.1 M)	0.001	30	14.4669	10.51	0.651	0.0296
	0.01	60	14.5346	12.7059	0.2345	0.0009
Phosphate buffer (0.025 M)	0	1	14.4438	6.5083	1.3211	0.0303
	0.0001	5	14.5196	6.7565	1.2685	0.1144
	0.001	30	14.4776	10.2455	1.1822	0.0565
	0.01	60	14.3654	12.4097	0.3546	0.0117
MQ	0	1	14.4333	2.4399	21.2418	0.0091
	0.0001	5	14.4592	3.1433	21.345	0
	0.001	30	14.6008	2.9699	21.1166	0.0881
	0.01	60	14.5826	6.9699	1.3047	0.0952
DIRO	0	1	14.6533	2.2812	21.2182	0.0164
	0.0001	5	14.4666	2.5701	21.2466	0.0116
	0.001	30	14.6125	6.2119	1.2313	0.0573
	0.01	60	14.468	9.9923	1.0777	0.0536
TAP	0	1	14.4174	3.4079	21.2142	0.0386
	0.0001	5	14.5883	3.2767	21.2839	0.0173
	0.001	30	14.5708	3.6175	21.4922	0.0173
	0.01	60	14.46	1.9172	21.1721	0.0216

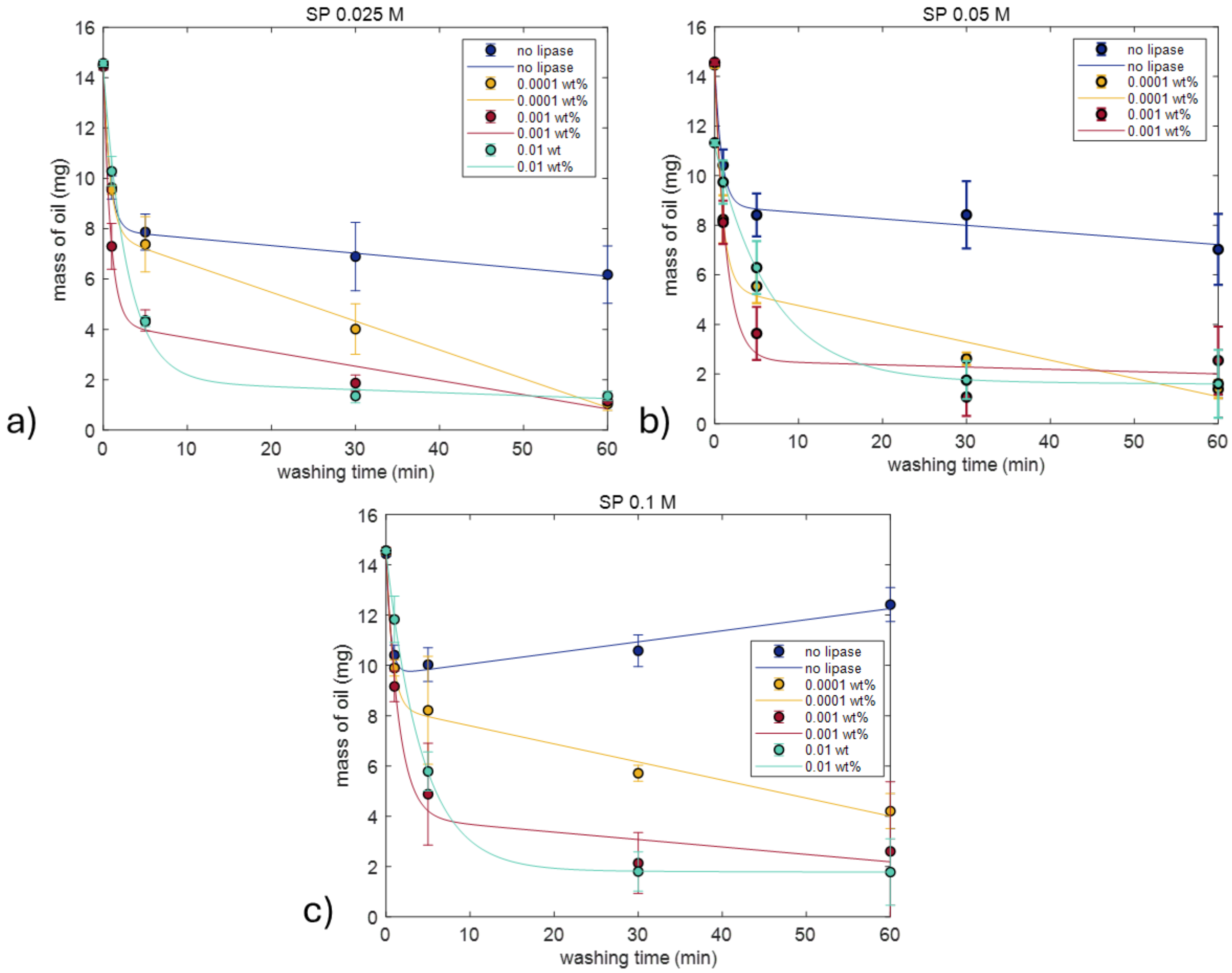

Figure S1: Average amount of olive oil remaining on the surface of plastic tubes (mg) after washing at 25 °C for 1, 5, 30, and 60 minutes using sodium phosphate buffer at two concentrations: (a) 0.025 M (b) 0.05 M (c) 0.1 M. Results are shown for conditions without lipase (blue) and with lipase (red, yellow, green) dissolved in the respective buffers. Each condition was tested in triplicate.

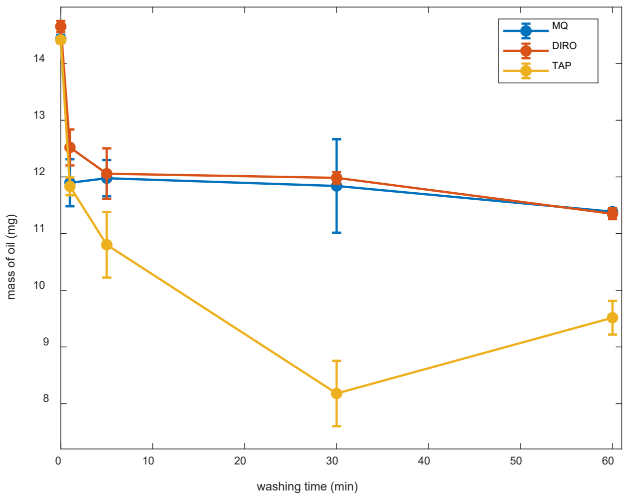

Figure S2: Average amount of olive oil remaining on the surface of plastic tubes (mg) after washing at 25 °C for 1, 5, 30, and 60 minutes using MQ, DIRO and TAP with no addition of lipase. Each condition was tested in triplicate.

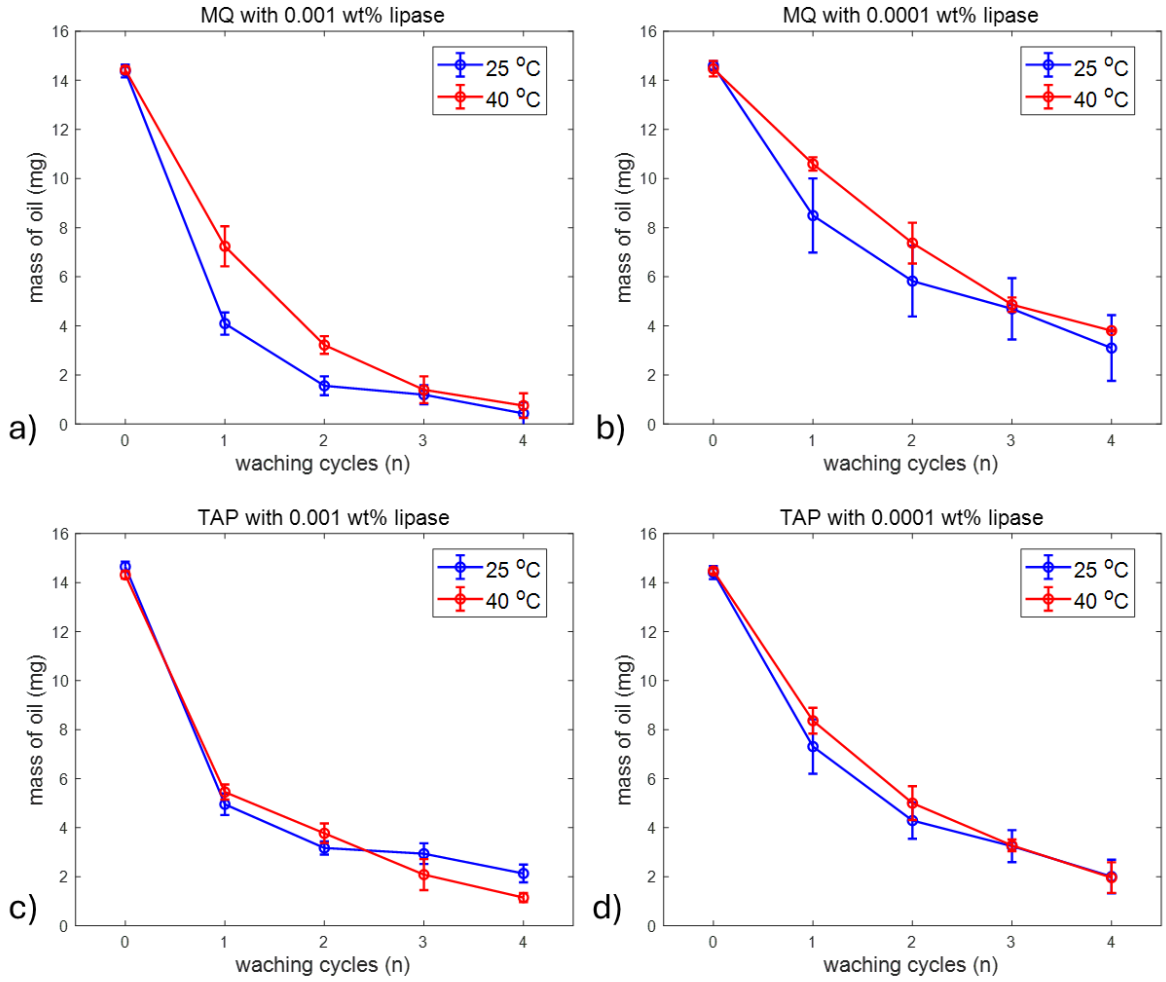

Figure S3: Comparison of the average amount of olive oil remaining on the surface of plastic tubes (mg) after washing at 25 and 40 °C for 1, 2, 3, and 4 washing cycles using MQ with (a) 0.001 wt% (b) 0.0001 wt% and TAP with (c) 0.001 wt% (d) 0.0001 wt%.

Table S4: Effects of time, number of washing cycles, lipase concentration and ionic strength on the oil removal from plastic surfaces using different solutions and water grades.

Solution/water grades	Time	Washing cycles	Lipase concentration	Buffer Ionic strength	T increase 25 to 40 °C

**Oil removal efficiency**	SP buffer	Increases	Increases	Increases	Increases	Constant
	Purified grades	Increases	Increases	Increases		Constant
	TAP	Constant	Increases	Decreases		Constant

1.3 QCM-D

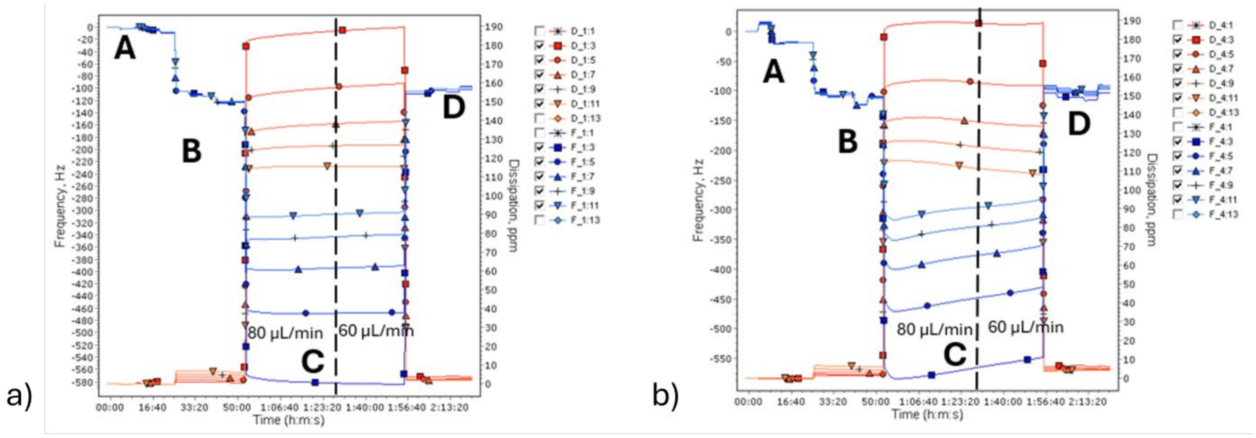

Figure S4: QCM-D results for DIRO water without lipase (a) and DIRO water with lipase (b). Frequency (blue) and dissipation (red) of overtones 3:11 are shown. The four regimes are: A - bare sensor in air, b -sensor coated with GDO in air, C - sensor with GDO in liquid, D – dried sensor after treatment with liquid. Experiments in air were repeated 5 times in air and all regimes are shown. The film thicknesses in the 2nd and 4th regimes are calculated by Voigt model.

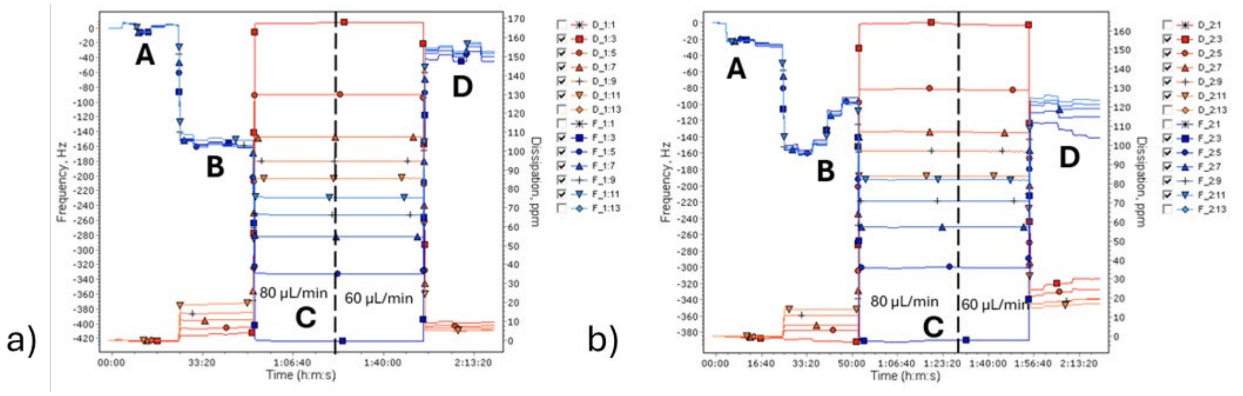

Figure S5: QCM-D results for TAP water without lipase (a) and TAP water with lipase (b). Frequency (blue) and dissipation (red) of overtones 3:11 are shown. The four regimes are: A - bare sensor in air, b -sensor coated with GDO in air, C - sensor with GDO in liquid, D – dried sensor after treatment with liquid. Experiments in air were repeated 5 times in air and all regimes are shown. The film thicknesses in the 2nd and 4th regimes are calculated by Voigt model.

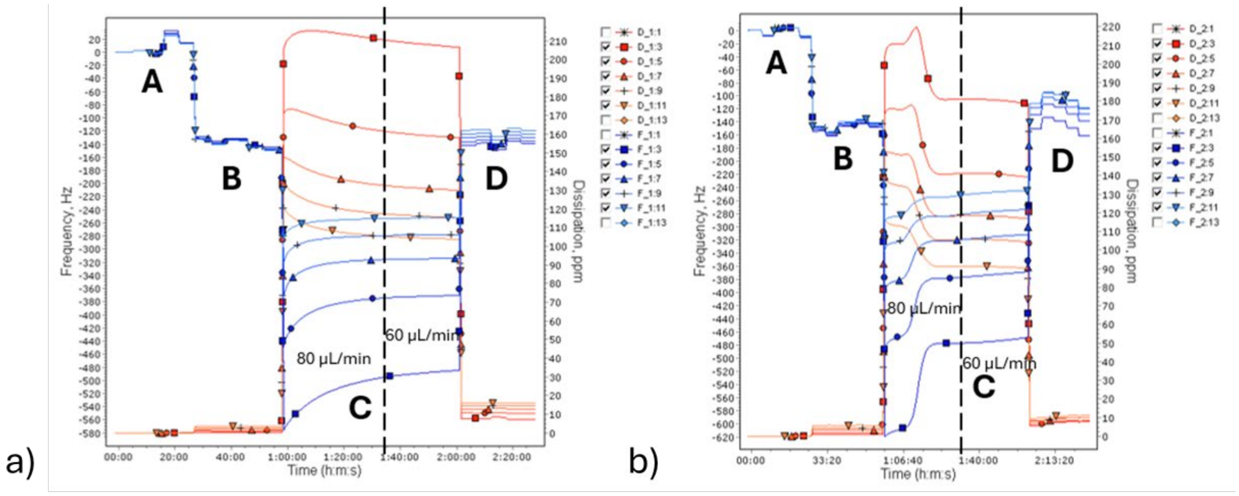

Figure S6: QCM-D results for sodium phosphate 0.025 M water without lipase (a) and sodium phosphate 0.025 M water with lipase (b). Frequency (blue) and dissipation (red) of overtones 3:11 are shown. The four regimes are: A - bare sensor in air, b -sensor coated with GDO in air, C - sensor with GDO in liquid, D – dried sensor after treatment with liquid. Experiments in air were repeated 5 times in air and all regimes are shown. The film thicknesses in the 2nd and 4th regimes are calculated by Voigt model.

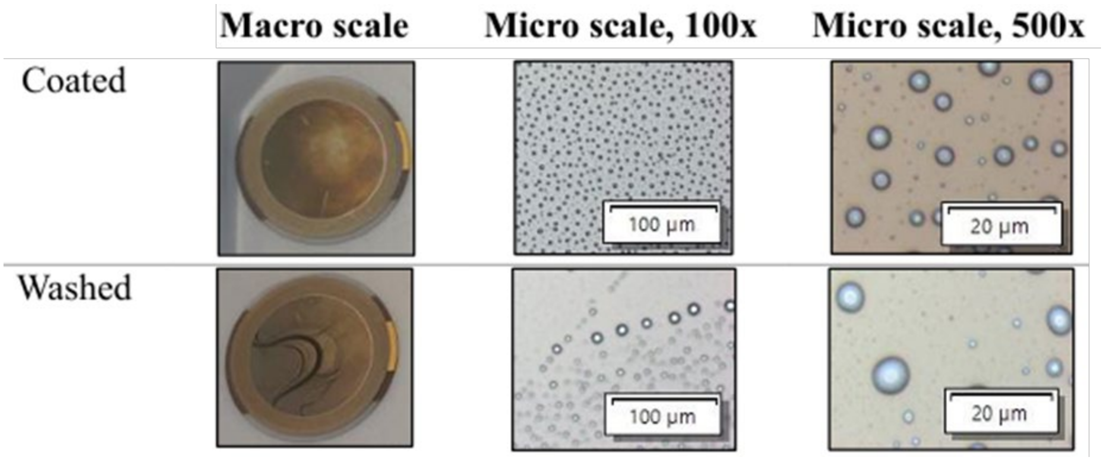

Figure S7: Example of coated and washed sensor after washing with MQ water. Both the coated and washing sensors were analysed on macro scale (images through a phone), and with the microscope in 100x and 500x magnifications. Scale bar is 100 and 20 µm respectively.

Table S5: Parameters used for modeling water grades and buffer with lipase in a coated senor. Water grades were modeled as bulk fluid. GDO corresponds to layer 1, lipase corresponds to layer 2.

Overtones 3-11 were used for modeling. The values shown for the layer thickness is the last value of the washing regime.

	Fixed parameters					Fitted parameters				Fitted parameters (vectors)	Fitted parameters (vectors)
Water grades	Fluid density (kg/m^3)	Fluid thickness (m)	Fluid viscosity (mPa s)	Layer 1 density (kg/m^3)	Layer 2 density (kg/m^3)	Layer 1 viscosity (mPa s)	Layer 1 shear (Pa)	Layer 2 viscosity (mPa s)	Layer 2 shear (Pa)	Layer 1 thickness (nm)	Layer 2 thickness (nm)
MQ	997	1	0.89	934	1350	0.4	27329	1.2	73860	0.3	23
DIRO	997	1	0.89	934	1350	0.8	24943	2.1	32118	6.6	27
TAP	997	1	0.89	934	1350	0.5	13317	0.9	1350	5	3.79
SP	997	1	0.89	934	1350	0.8	30167	1.4	1350	1	46

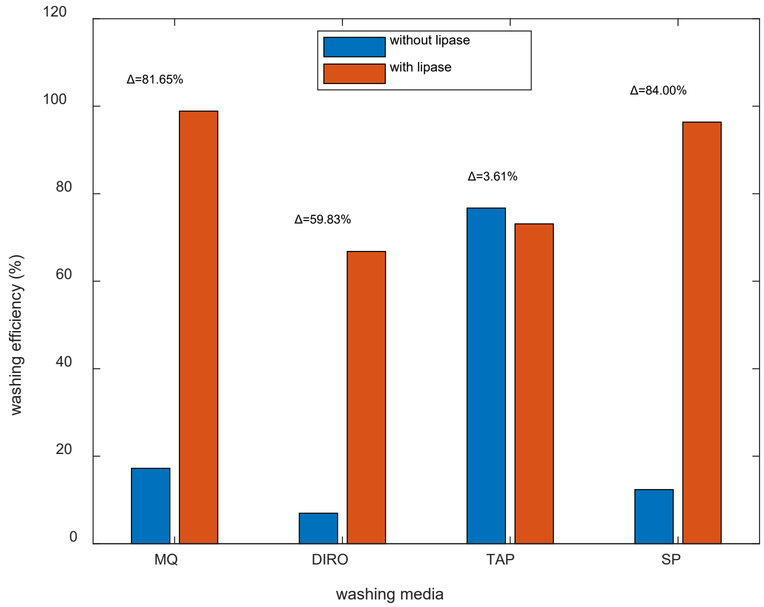

Figure S8: Washing efficiency of removing DGO from a silica surface (blue) without any lipase (orange) with lipase. Δ is to the washing efficiency difference when washing with /without lipase with the same media for 1 hour. Here, the thickness of layer 1 from table S4 is used as the final thickness for the GDO layer. The thickness of the film was measured with the Voigt model both before and after washing.

Table S6: Calculated thickness of GDO thin film before and after water treatment. In all cases Voigt model was used.

Film thickness (nm)

	In air before washing	In air after washing
MQ	26.7	22.1
MQ with lipase	26.9	14.3
DIRO	20.1	18.7
DIRO with lipase	19.9	18
TAP	30.45	7.09
TAP with lipase	18.6	18
SP 0.025 M	28.3	24.8
SP 0.025 M with lipase	27.6	25.7
MQ with 2 wt% SDS	34	3.5

1.4 Contact angle measurements

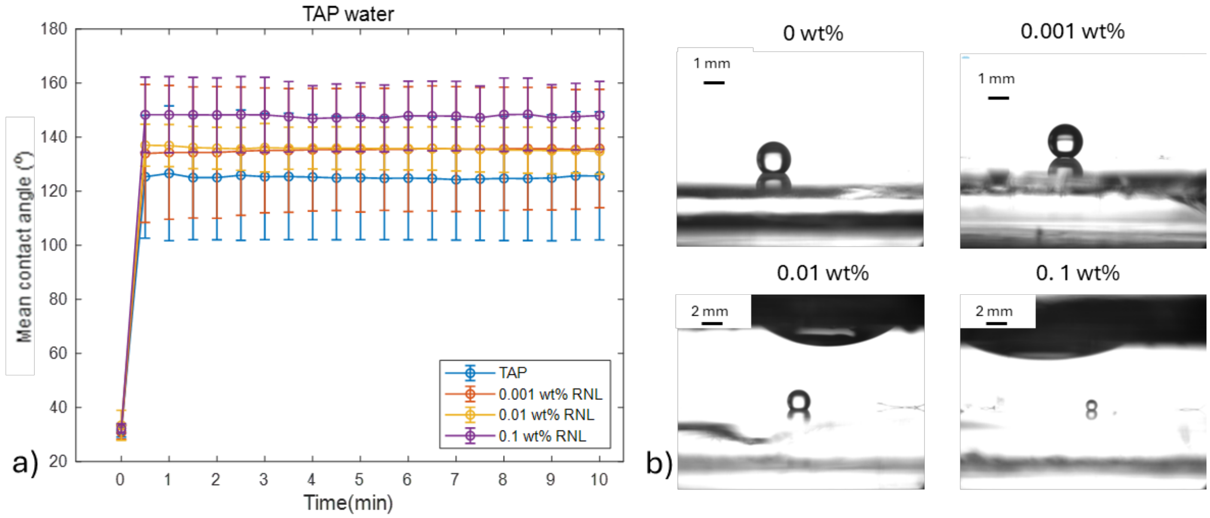

Figure S9: a) Contact angle measurements of olive oil in TAP water with and without addition of lipase, on glass surface over time. The contact angle of olive oil (º) is plotted as a function of time (min). One measurement was taken every 30 seconds for a total of 10 minutes. b) olive oil droplets in water with different lipase concentrations.

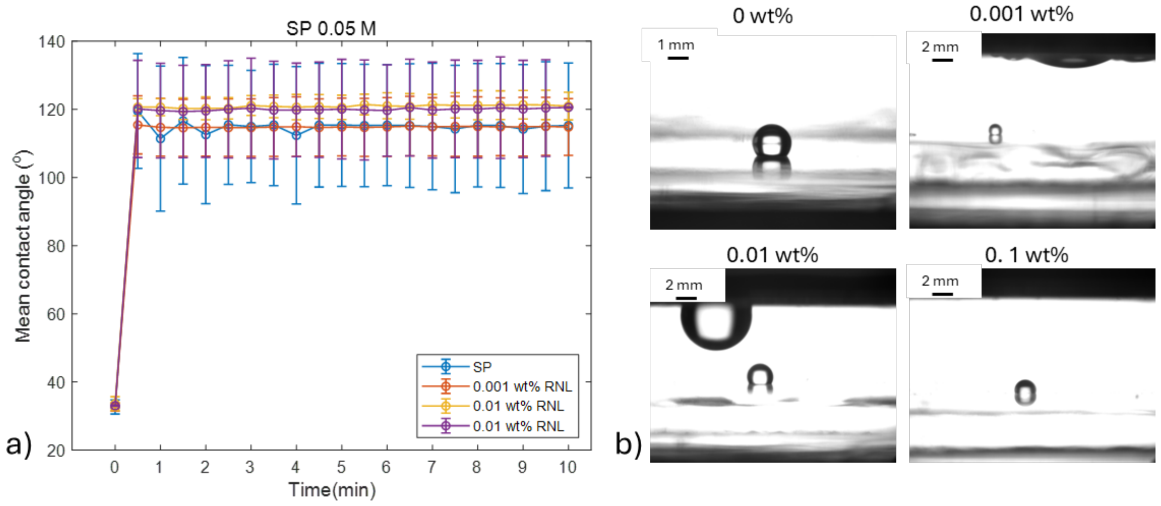

Figure S10: a) Contact angle measurements of olive oil in sodium phosphate buffer 0.05 M with and without addition of lipase, on glass surface over time. The contact angle of olive oil (°) is plotted as a function of time (min). One measurement was taken every 30 seconds for a total of 10 minutes. b) olive oil droplets in water with different lipase concentrations.

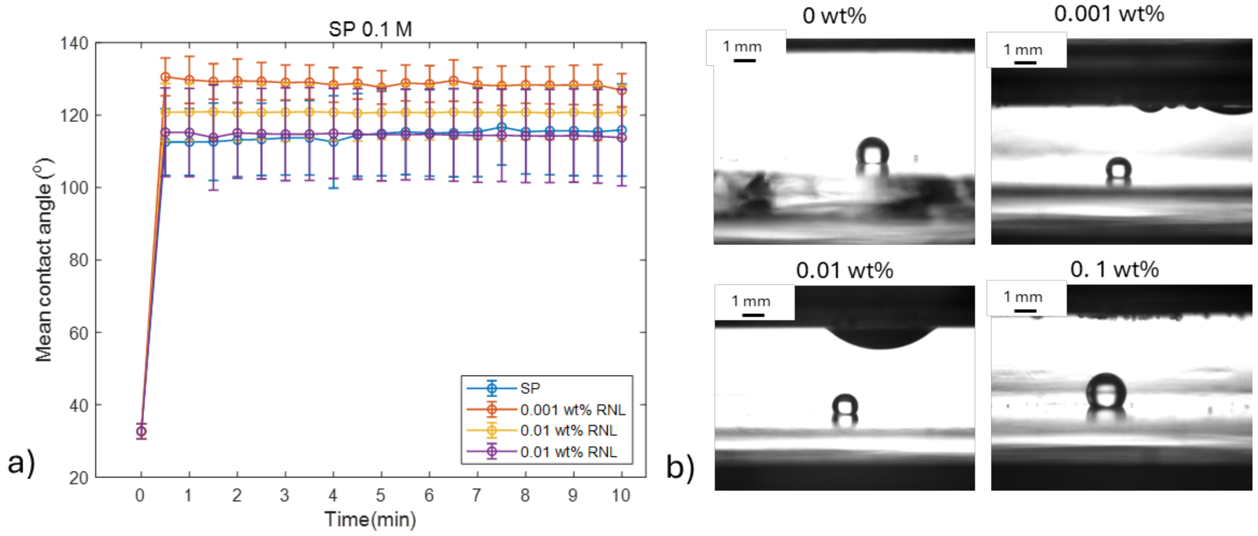

Figure S11: a) Contact angle measurements of olive oil in sodium phosphate buffer 0.1 M with and without addition of lipase, on glass surface over time. The contact angle of olive oil (°) is plotted as a function of time (min). One measurement was taken every 30 seconds for a total of 10 minutes. b) olive oil droplets in water with different lipase concentrations.

The contact angle of all the solutions was measured in air on a plastic and glass surface. Across all six solvents/waters, the measured static contact angle on a plastic surface has a wide window of

values ranging from 68 ° to 110 ° (Table 2). These values indicate, as expected, that none of the buffers and water wet the plastic surface.

Table S7: Contact angle measurements of different buffer concentration and water grades without and with addition of RNL, on a plastic surface. Young Laplace was used to estimate the angle.

Lipase (wt%)	SP 0.1 M	SP 0.05 M	SP 0.025 M	Milli-Q	DIRO	TAP
0	68.06	88.78	96.80	88.78	90.85	99.38
0.001	88.09	91.87	100.42	94.23	90.01	107.87
0.01	92.36	102.19	104.31	71.76	91.23	108.77
0.1	89.41	112.84	104.13	74.42	95.61	96.04

The contact angle of the olive oil could not be measured as it spread on the surface.

1.5 Interfacial tension

The following interfacial tensions were measured:

1) Oil in air
2) Oil in water
3) Oil in water with 0.1 wt% lipase
4) Water with 0.1 wt% lipase in air

Below some of the frames are shown:

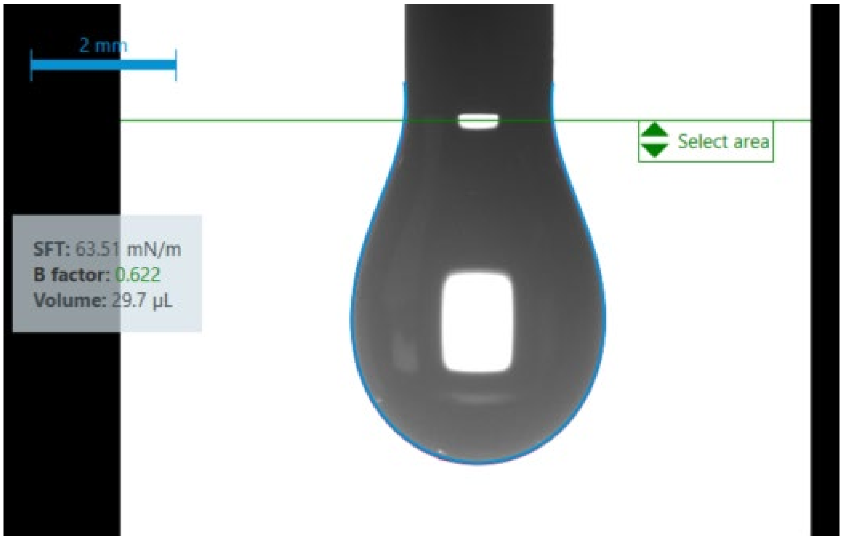

Figure S12: Surface tension of water with 0.1 wt% lipase in air. Needle diameter is 1.5 mm.

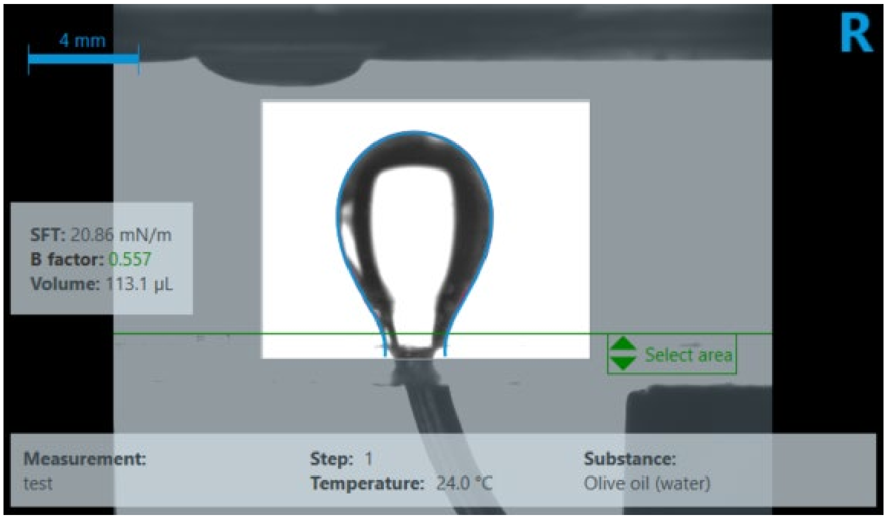

Figure S13: Interfacial tension of olive oil in water. Tube diameter is 1.5 mm.

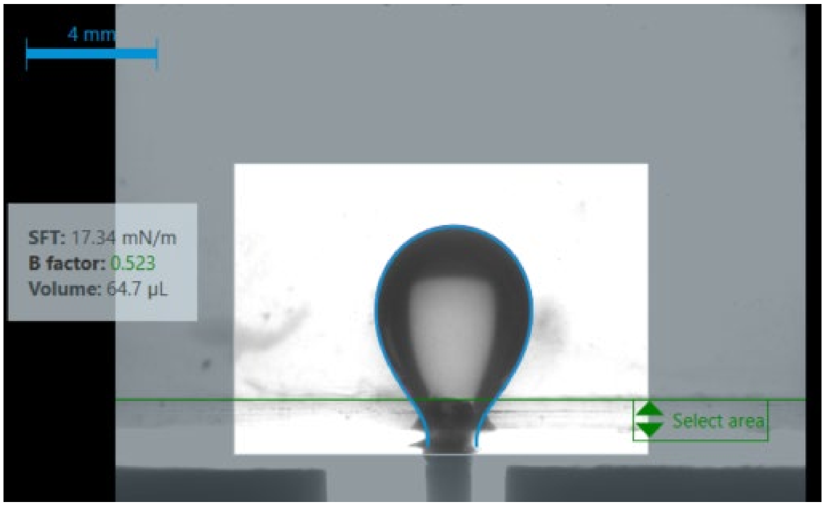

Figure S14: Interfacial tension of olive oil in water with 0.1 wt% lipase. Tube diameter is 1.5 mm.

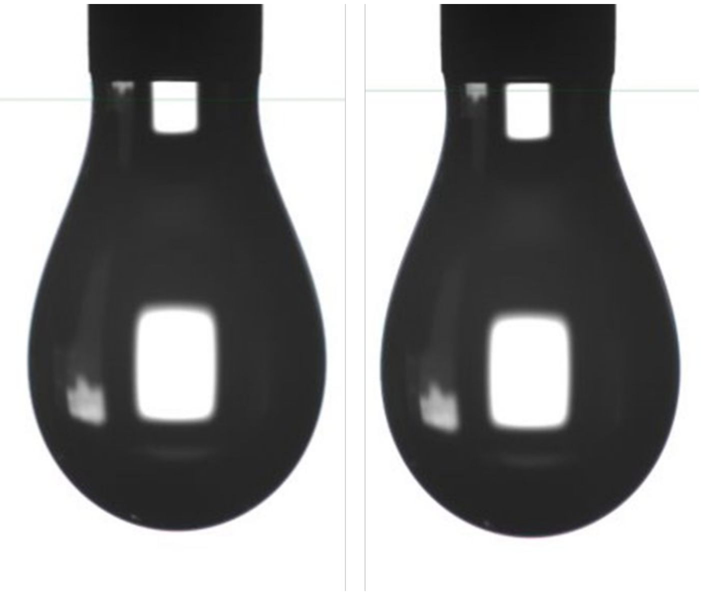

Figure 15: Surface tension of water in air. Needle diameter is 2mm. The difference between the two figures is that the green line (which is the line taken as the beginning of the droplet) is slightly shifted. This small change alter the surface tension up to 2 mN/m.

Figure 16 shows that calculating the surface tension via the drop shape method can be a delicate process as various parameters can alter the final surface tension value. First, the needle diameter should be the optimal so as the liquid in question, to form the correct shape. Second, the contrast should be accurate, so the software is able to detect in detail the outline of the drop. Finally, the beginning of the drop should be carefully placed (the green line) to obtain a correct value. In our data we chose to add the value of 69.2 for the surface tension of water/air as it is the mean value obtained both from the drop shape analyzer as well as the drop volume apparatus. Although it is known that the surface tension is 72 mN/m we decided to follow the experimental data that we obtained.

References

(1) Tsompou, A.; Kocherbitov, V. Surface and bulk mechanisms in repeating treatment of solid surfaces by purified water. *Heliyon* **2023**, *9* (6), e17163. DOI: https://doi.org/10.1016/j.heliyon.2023.e17163.
(2) Tsompou, A.; Kocherbitov, V. Optimizing mild surface cleaning methods: influence of water purity and pH. *Scientific Reports* **2025**, *15* (1), 29815.
